# Azure-winged magpies’ decisions to share food are contingent on the presence or absence of food for the recipient

**DOI:** 10.1038/s41598-020-73256-0

**Published:** 2020-09-30

**Authors:** Jorg J. M. Massen, Sofia M. Haley, Thomas Bugnyar

**Affiliations:** 1 Animal Ecology Group, Department of Biology, Utrecht University, Utrecht, The Netherlands; 2 Department of Behavioral and Cognitive Biology, University of Vienna, Vienna, Austria; 3 Department of Biology, Lund University, Lund, Sweden

**Keywords:** Anthropology, Social evolution, Animal behaviour, Behavioural ecology

## Abstract

Helping others is a key feature of human behavior. However, recent studies render this feature not uniquely human, and describe discoveries of prosocial behavior in non-human primates, other social mammals, and most recently in some bird species. Nevertheless, the cognitive underpinnings of this prosociality; i.e., whether animals take others’ need for help into account, often remain obscured. In this study, we take a first step in investigating prosociality in azure-winged magpies by presenting them with the opportunity to share highly desired food with their conspecifics i) in a situation in which these conspecifics had no such food, ii) in a situation in which they too had access to that highly desired food, and iii) in an open, base-line, situation where all had equal access to the same food and could move around freely. We find that azure-winged magpies regularly share high-value food items, preferably with, but not restricted to, members of the opposite sex. Most notably, we find that these birds, and specifically the females, seem to differentiate between whether others have food or do not have food, and subsequently cater to that lack. Begging calls by those without food seem to function as cues that elicit the food-sharing, but the response to that begging is condition-dependent. Moreover, analyses on a restricted dataset that excluded those events in which there was begging showed exactly the same patterns, raising the possibility that the azure-winged magpies might truly notice when others have access to fewer resources (even in the absence of vocal cues). This sharing behavior could indicate a high level of social awareness and prosociality that should be further investigated. Further studies are needed to establish the order of intentionality at play in this system, and whether azure-winged magpies might be able to attribute desire states to their conspecifics.

## Introduction

Helping non-related individuals is considered to be one of the pinnacles of human behavior^1^. Due to its counterintuitive nature with regard to natural selection, helping behavior has sparked inquiry into phylogenetic history and has consequently inspired studies on other animal species over the past couple of decades. Although human helping is still considered unparalleled in degree^1,2^, evidence for helping / prosocial behavior in other animals, here defined as voluntary actions that benefit another individual at no or low costs to the actor^3^, is increasing and comprises reports on our closest living relatives (bonobos, *Pan paniscus*^4^; chimpanzees, *Pan troglodytes*^5^; but see^6,7^ for a discussion of chimpanzee prosociality), other nonhuman primates (reviewed in^8^), other social mammals (e.g. rats, *Rattus norvegicus*^9^; dogs, *Canis lupus familiaris*^10^) and birds (pinyon jays, *Gymnorhinus cyanocephalus*^11^; azure-winged magpies, *Cyanopica cyanus*^12^; African Grey Parrots, *Psittacus erithacus*^13^). The wide variety of paradigms and procedures used, however, can inhibit inferences about the evolutionary history of helping (but see^14^). Moreover, many of these studies rely on relatively artificial experimental set-ups that may not accurately reflect situations and environments that the animals would encounter naturally.

Food-sharing is one of the helping behaviors that can, in fact, be seen in the natural world. Defined as “the unresisted transfer of food from one food- motivated individual to another”^15^, food-sharing among humans can be observed at daily-life events such as dinner parties, in contemporary forager societies^16,17^; and there is evidence suggesting that it was already present in pre-historic humans^18^. Food-sharing among non-human animals is also relatively common, yet mostly concerns sharing food with related offspring; i.e. parental care^19^, which can be explained by kin-selection^20^. In contrast, food-sharing among adults is relatively rare, although not absent. As with prosociality in general, recent studies have reported adult-adult food-sharing in our closest living relatives (bonobos even between groups^21^, but see^22^; chimpanzees^22–24^, other nonhuman primates (reviewed in^25^), other social mammals (e.g. vampire bats, *Desmodus rotundus*^26^; killer whales, *Orcinus orca*^27^) and several bird species^28–34^.

Apart from its evolutionary significance, food-sharing as a prosocial behavior has also gained interest due to the apparent cognitive requirements of this behavior^35^. Traditionally, food-sharing was of interest due to its possible reciprocal nature (e.g.^22,26,30,36,37^) and the cognitive capacities this may or may not require^38–42^. Lately, food-sharing from parents to offspring has been considered important in facilitating learning about what is edible and how to process food, and might under certain circumstances even be regarded as teaching (for a review see^35^). Most notably, however, some researchers have adopted food-sharing paradigms to inquire whether animals can understand the needs of others and/or even attribute desire states to others; i.e. do they understand that others want the food they are sharing?

Chimpanzees, for example, distinguish whether their conspecifics are with or without food, and appear to only help those who are without^43^. Moreover, they seem to have some understanding of their conspecific’s goals as they can flexibly adjust their helping behavior to the specific task their conspecific is engaged in^44^. Partners may use begging or specific requests as cues to communicate their needs and/or desires. Findings on the mediating role of active requests of the recipients in chimpanzee prosociality, however, are rather inconsistent, with reports of positive effects on targeted helping^24,43,45^ and negative effects on prosocial behavior in both a prosocial choice task^46^ and a food-sharing task^47^. To examine whether prosocial food-sharing is motivated by sympathy, Liebal and colleagues^48^ adapted an existing testing paradigm for children^49^, to investigate whether apes are more likely to help a conspecific after it was ‘harmed’. Interestingly, neither chimpanzees nor the other African apes showed such sympathetic concern, and only orangutans helped their conspecifics more after they had been harmed than when not^48^. Consistent with these findings, orangutans also seem to share food more actively than chimpanzees do^50^, and show flexible targeted helping in a token exchange task^51^.

Some of the most convincing studies investigating desire attribution while sharing food, however, come from birds. In an elegantly designed experiment, Ostojić and colleagues^31^ showed that male Eurasian jays, *Garrulus glandarius*, specifically fed their mates food that they had not previously eaten and avoided sharing the food their mates had been sated on, regardless of what the males themselves had experienced^31^. These findings are particularly significant as they show that the subject differentiates its own desires from that of another; i.e., would meet the criteria of second order intentionality^52^. A recent study on New Zealand robins, *Petroica longipes,* replicated these findings in the wild showing that these males also cater to their mate’s desires by providing their mates with food they had not previously eaten. This concurred with separate results indicating the females’ preferences for variety^33^.

When studying food-sharing, it is important to distinguish passive food-sharing from active food-sharing. The former comprises co-feeding and so-called ‘tolerated theft’, and inferences about the motivations of the ‘actor’ are very difficult to make. With regard to active food-sharing, it is also important to distinguish sharing-under-pressure^53^ from sharing as a free choice. Sharing-under-pressure is most likely a harassment avoidance behavior^15^ and as such can be considered a mutualistic interaction^54^ in which the actor caters to its own needs (i.e., avoiding harassment), rather than specifically addressing (or having knowledge of) the needs of the others. Finally, in light of the different orders of intentionality^52^, it is important to distinguish reactive from proactive food-sharing; i.e. with or without request respectively.

In this study, we investigated whether azure-winged magpies (*Cyanopica cyanus*) take the availability of food to their conspecifics into account when making decisions about sharing food, while also considering a potential cue for the lack of food of conspecifics; i.e. begging. The azure-winged magpie is a corvid species native to Eastern Asia. Azure-winged magpies have a cooperative breeding lifestyle in which related as well as unrelated individuals help out at the nest, and food-sharing can be observed between parents and offspring, and between both related and unrelated adults^55–57^. Moreover, a recent experimental study showed that azure-winged magpies show high levels of proactive prosociality^12^. Furthermore, azure-winged magpies show elaborate physical- and socio-cognitive skills, like solving, and partially understanding the string-pulling task^58^, and being able to make transitive inferences^59^.

We tested 10 azure-winged magpies on how proactively they shared highly valued mealworms with their conspecifics in a situation in which these conspecifics had no access to mealworms themselves, in a situation in which the conspecifics had access to the same amount of mealworms as the focal individual had, and in a situation in which all individuals were in the same compartment and had similar access to mealworms. Moreover, we recorded all vocalizations to infer whether any potential food-sharing was requested or caused by vocal cues. We expected that there should be more begging by the conspecifics when they had no food, and that the amount of begging might predict whether or not sharing occurs.

## Materials and Methods

### Subjects

Subjects were 10 adult azure-winged magpies (4 males, 6 females; age range when tested = 1.5 – 6 years of age; see Table 1) living at the Animal Care Facilities of the Department of Cognitive Biology of the University of Vienna. The birds live in two separate social groups of four (group 1: 2F & 2 M) and six (group 2: 4F & 2 M) individuals in two outdoor aviaries (6 m x 3 m x 3 m and 4.25 m x 3 m x 3 m respectively) with roof covering. These aviaries contain living plants, swings and sitting branches as well as several enrichment objects. The birds are fed twice a day with a mixture of fruits, seeds, insects, and commercially available bird food, and occasionally also get meat or eggs. The birds were never food-deprived. On days of an experiment, however, they did not receive mealworms (which are a highly preferred food item) in the morning (see manipulations below), but did receive other (and enough) food. Water for drinking and bathing was available ad libitum in all compartments.

**Table 1:** Test subjects, sex, year of birth (YoB), breeder status (*BR* breeder, *NB* non-breeder), group.

Name	Sex	YoB	Breeder status	Group	Group created*
Han Solo	Male	2014	BR	1	Nov. 2014
Leia Organa	Female	2014	BR	1	
Amidala Naberrie	Female	2012	NB	1	
Jabba the Hutt	Male	2015	NB	1	
Chewie	Female	2015	NB	2	Nov. 2016
Anakin Skywalker	Male	2015	NB	2	
BB8	Female	2016	NB	2	
Poe Dameron	Female	2016	NB	2	
Kylo Ren	Male	2016	NB	2	
Rey	Female	2016	NB	2	

*Note that if the birth year is later than the date the group was started, the individual was born into this group.

### Ethical note

This study was non-invasive, and all birds participated on a voluntary basis; i.e. whenever one of our birds showed any signs of distress the experiment was aborted and all birds were reunited with each other. As a consequence, the study complied with Austrian law and adhered to the Guidelines for the treatment of animals in behavioral research and teaching^60^ of the Animal Behavior Society (ABS). Moreover, the design of this study was approved by the Animal Ethics and Experimentation Board of the Faculty of Life sciences, University of Vienna (case number: 2018–015). After the study, all birds remained in captivity at the Animal Care Facilities of the Department of Cognitive Biology at the University of Vienna.

### Test-procedure

**Figure 1:**
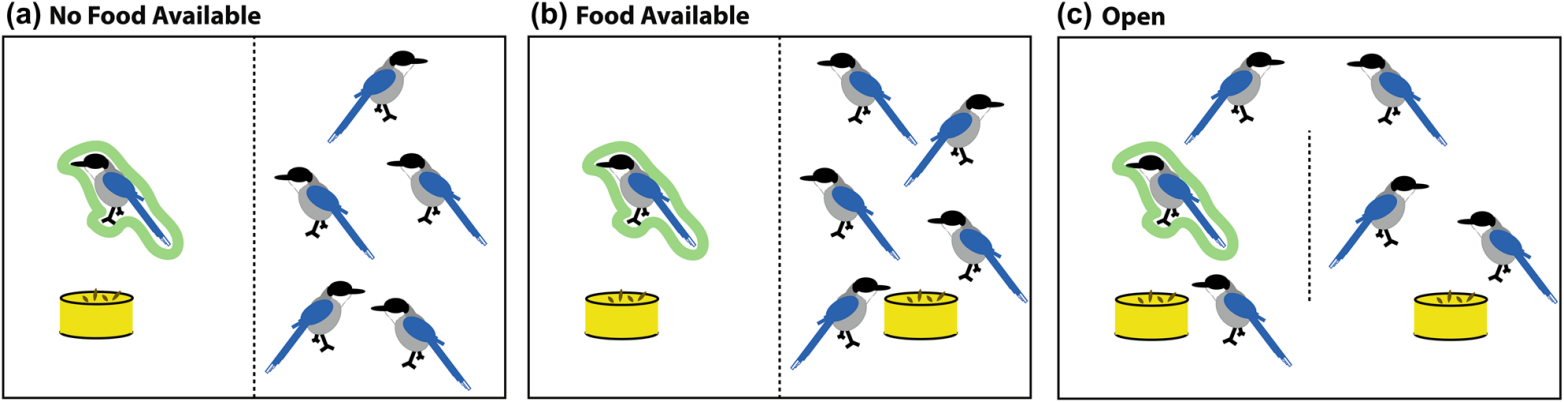
Schematic of the test conditions: (**a**) the no food available condition, (**b**) the food available condition, and (**c**) the open condition. The focal bird is circled in green. Here as an example 6 birds, like in group 2 of our population.

The experiment was conducted between April 24th 2018 and July 11th 2018. In this experiment all birds were subjected to three different conditions (1 per session) twice in 2 different rounds (Fig. 1). Order of conditions was counterbalanced over subjects and rounds. Each session lasted 15 min. The conditions were as follows:

*The No Food Available Condition:* Prior to the start of a session in this condition the subject was separated from the rest of the group into a compartment of the home-enclosure (for both groups these compartments were 2 m x 3 m x 2 m in size). Separation was on a voluntary basis; i.e. we lured subjects into this compartment by positive reinforcement, and would immediately abort a session if any bird showed signs of distress due to this separation. Notably, the walls between the different compartments are wire-mesh, and thus the birds were still in visual and auditory contact with each other. Moreover, they were also able to potentially share food through the wire-mesh partition.

After separation, the birds were given 2 min to acclimatize to the new situation. After that the subject was provided with a bowl containing 15 g of mealworms, whereas in the compartment of the rest of the group an empty bowl was placed; i.e. they did not receive any mealworms (see Fig. 1a). Note that 15 g is about half of what we normally feed the whole group and thus clearly enough for one adult azure-winged magpie (weighing approximately 76 – 118 grams^61^).

*The Food Available Condition:* This condition was the same as the No Food Available Condition, apart from the fact that in this condition the other birds of the group were also provided with one bowl containing 15 g of mealworms in their compartment, thus creating a situation in which there was little need for the subject to share its mealworms with its group mates as they had access to mealworms themselves (see Fig. 1b). Note again that this amount of mealworms is about half of what we normally feed the whole group, and thus more than enough for all other birds. Note also that azure-winged magpies tend to behave very tolerantly at food sources (see^12^), and consequently we never witnessed any bird monopolizing the food bowl.

*The Open Condition:* This condition aimed at capturing ‘base-line’ frequencies of sharing behavior in a ‘normal’ feeding situation and additionally explored the potential effect of the separation in the other two conditions as here no bird was separated. As in the *Food Available Condition,* two bowls, each containing 15 g of mealworms, were placed in the two different compartments of the birds’ cage that were used for the test, yet now all birds had access to both bowls (see Fig. 1c). Per session of the Open Condition we focused on (through focal follows) the sharing behavior of one individual; i.e. like the No Food Available and Food Available conditions, we conducted 2 sessions of the Open condition per animal, and thus a total of 20 of such sessions.

Using a handheld camcorder (Panasonic HC-X909) SMH made focal recordings of our subjects. While recording, she spoke into the microphone of the camera, thereby recording live the behaviors she observed. Additionally, videos were analyzed post-hoc using Solomon coder beta v. 12.09.02 (Péter, 2012). In addition to the handheld, another camcorder (Canon-LEGRIA HF-G25) was placed outside one of the corners of the compartment of the potential receivers with an angle covering that whole compartment. Finally, a directional microphone (RØDE NTG-1) with windshield and connected to a Zoom H4n Pro audio recorder was placed next to that second camcorder in order to record the vocalizations of the potential receivers.

We specifically coded the instances of active sharing between the subject and a group-member, and defined this operationally as giving mealworms to another individual through beak-to-beak contact. Second, we measured all instances of passive sharing, operationally defined as letting another bird take food without actively offering (i.e., giving or actively directing mealworms towards another individual) or protesting (i.e., moving worms away from other individual and showing signs of distress if other individual takes food). Third, we measured the time the birds spent caching mealworms; i.e., purposefully leaving or hiding food. Finally, from the audio-recordings, we measured the number of specific vocalizations of the non-focal birds, and where possible from each specific bird. We paid specific attention to begging behavior; i.e., soft, high-pitched peeping calls accompanied by following behavior directed towards the individual that has food. We measured these behaviors from the audio recordings as our videos did not reliably depict in detail what the potential recipients were doing (apart from the audible begging), and consequently we could also not analyze whether the begging of a specific individual led to sharing with that specific animal. For a full ethogram of all other behaviors and vocalizations coded, please see the supplementary information. SMH coded all videos, and JJMM recoded a random selection of 15% of those videos to test for inter-rater reliability. As the context of each condition was clear at the start of each video; i.e. whether doors where open or closed or whether conspecifics where given food too, neither coding nor recoding could be performed blindly. Nevertheless, reliability of coding active sharing was high (Spearman’s rho = 0.89, n = 9, *p* < 0.01; agreement: 83.3%). Passive sharing, begging and caching occurred too seldomly across the different conditions to perform formal statistics though agreement was very high (passive sharing: 89%) to perfect (both begging and cashing: 100%).

### Additional measures

To investigate whether food-sharing was selective or generic, we examined the relationships between the group members prior to our experiments, and analyzed whether food-sharing was dependent on relationship quality. As a measure of relationship quality we used tolerance for proximity at a food source^62^. To that effect, we performed 18 sessions of a social tolerance experiment between December 13th 2017 and May 23rd 2018. In these experiments, two mealworms were attached to two separate strings, 30 cm apart, which were on a platform outside their cage. The azure-winged magpies could access these strings and pull the mealworms into reach for consumption. Per session, 20 of such trials were conducted with an inter-trial interval of 20 s. We measured which birds were sitting next to each other at the perch in front of the platform, and the total of such tolerated proximity was used as measure of inter-individual relationship quality (cf.^63^).

### Analyses

Food-sharing, as well as begging data was heavily inflated by zeros. Therefore, we decided to analyze these data using a hurdle approach, where we first (hurdle 1) modeled what could influence the likelihood of sharing (y/n; with all ≥ 1 as yes) using a binomial generalized linear mixed model with a logit link function, and second (hurdle 2), if there was food-sharing (i.e. only data ≥ 1), what would influence the number of food-sharing events using a negative binomial generalized linear mixed model with a log link function, both using the lme4-package^64^ in R^65^. In these models we entered individual ID nested in group as random effects, and condition and sex as well as the interaction between condition and sex as fixed effects. In addition we ran models on food-sharing that included begging in order to see whether there was a direct link between these parameters. We also looked at the interaction between begging and condition, to see whether a potential link would be influenced by the different conditions.

Due to technical problems, unfortunately, the handheld camcorder did not record and save all experimental sessions; i.e. we did not have a record of 2 sessions (one in the Food Available Condition and one in the Open Condition), and recorded no sound in one session (restricting the analyses of vocalizations for that session (see data)). Furthermore, in one session (in the Open Condition) the camera saved only part of the session, and we needed to abort one session ~ halfway due to heavy rain. Whereas the former were treated as missing data, for the latter we added per session its duration and added this duration as a weight to our models.

To analyze whether the azure-winged magpies are selective as to with whom they share their food, we structured our data in a binary way, and noted down per session whether the subjects shared (y/n) with either of their group-members. We analyzed these data using a binomial generalized linear model with a logit link function using lmer4. Again we nested individual ID in group as random factors. We added our measure of social tolerance between the two birds and the sex-combination of the two birds (cf.^66^), as well as the interaction between sex-combination and social tolerance as fixed factors. Unfortunately, the quality of the videos we made did not allow us to assign begging calls to specific individuals, thus restricting our analyses of begging to the (recipient) group level only.

We compared all our models with their respective null models (i.e. including only the random (and control) effects), and address specifically when the test model did not deviate from the null model. We set alpha at 0.05 for all analyses and we used Tukey adjustments for post-hoc comparisons, and we thus report adjusted *p* values for such post-hoc comparisons. For model summaries and R-code please see the supplementary information.

## Results

### Sharing

**Figure 2:**
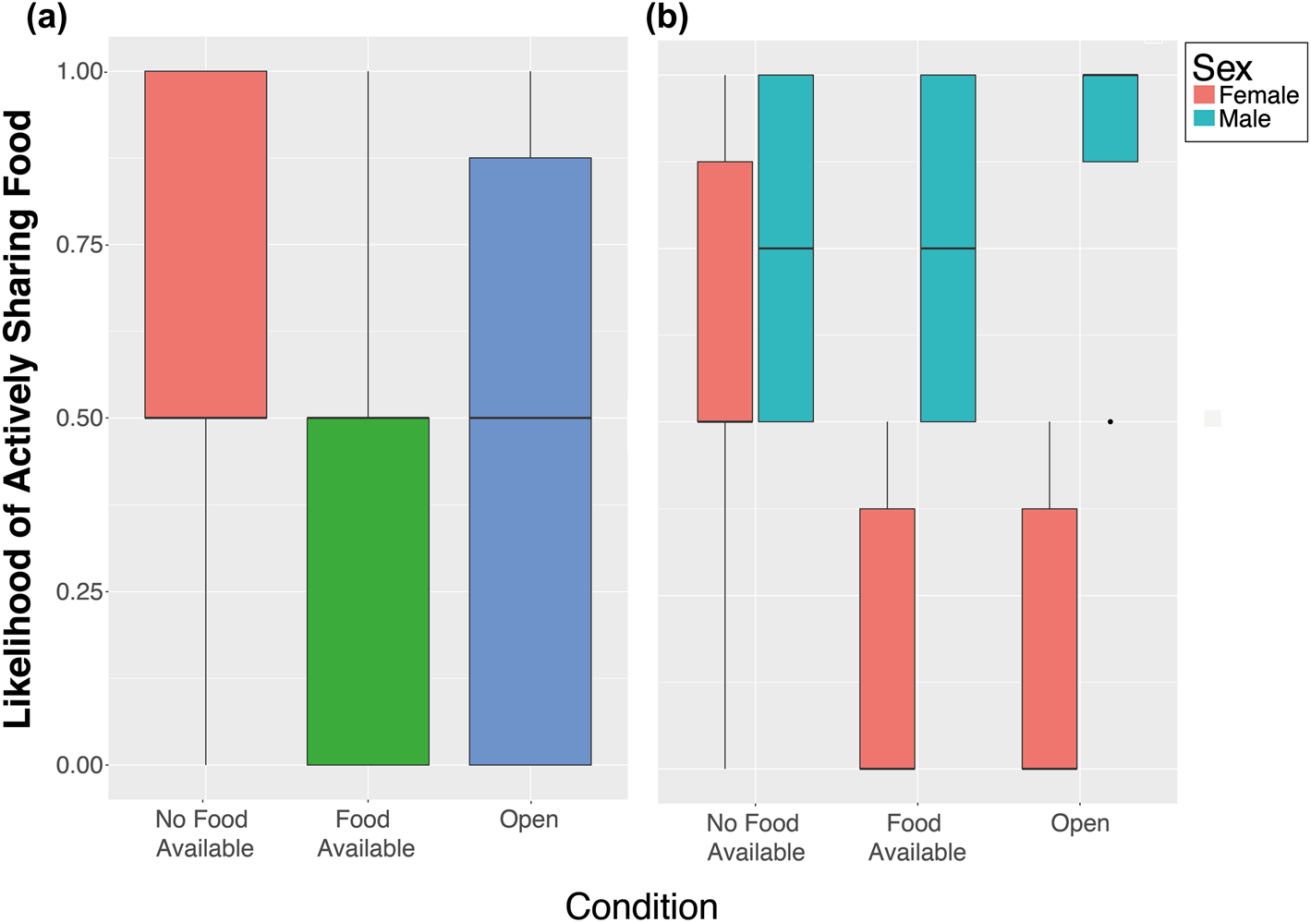
Likelihood of actively sharing mealworms by the subjects (**a**) when their conspecifics did not have access to mealworms (no food available), when they also had access to mealworms (food available) or in a situations in which all birds had similar access to all compartments as well as to mealworms, and (**b**) split up for the different sexes. Graphs show median (solid line), 25th and 75th percentile (box) and the largest and smallest value within 1.5 times the interquartile ranges respectively (whiskers).

The 15 g of mealworms we provided our subjects with was clearly enough for them to satiate on, and the birds spent on average 5.30% ± SEM 1.33 of their time caching the mealworms for later consumption. We found, however, no significant differences in the amount of time birds spend caching between the different conditions (LMM: F_2,44_ = 2.05, *p* = 0.14), nor sex differences or an interaction between sex and condition (see SEM). Apart from birds caching surplus mealworms, we also witnessed a total of 109 food-sharing events from nine of the ten subjects to a conspecific during the experiments (Mean/session = 1.87 ± SEM 0.47). The majority of these food-sharing events (i.e. 92 = 84%) were active food-sharing events where the focal subject proactively gave mealworms to another individual through beak-to-beak contact. In contrast, passive sharing occurred when the focal bird let another bird take food from them without actively offering or protesting, which in the No Food Available and Food Available Condition nonetheless required the subject to move towards the mesh partition with the food in its beak. Given the low occurrence of this behavior however, we did not perform any analyses on passive sharing, and only analyzed active sharing and total sharing (i.e. both active and passive sharing lumped). Results for analyses on total sharing and active sharing only were similar and therefore here we only report analyses on active sharing. For the results on total sharing, please see the Supplementary Information. When considering active food-sharing only, we found a strong effect of condition, namely that the birds were more likely to actively share food in the No Food Available Condition compared to the Food Available Condition (Estimate = 2.46, z = -8.170, *p* < 0.001), as well as when compared to the Open Condition (Estimate =—2.51, z = -8.362, *p* < 0.001)(Fig. 2a). Additionally, we found an interaction between condition and sex, which showed that whereas females seemed to differentiate between the conditions in which the other birds had a need or not (No Food Available vs Food Available: Estimate = 2.46, z = 8.170, *p* < 0.001; No Food Available vs. Open: Estimate = 2.50, z = 8.362, *p* < 0.001; Fig. 2b), males did not show any significant differences between the conditions, suggesting that only females considered the availability of food to their conspecifics when actively sharing food. When considering the actual number of active sharing events in those sessions in which active food-sharing happened (second hurdle) we found a similar but non-significant pattern and the full model did not differ significantly from the null model (see Supplementary Information).

### Sharing with whom

**Figure 3:**
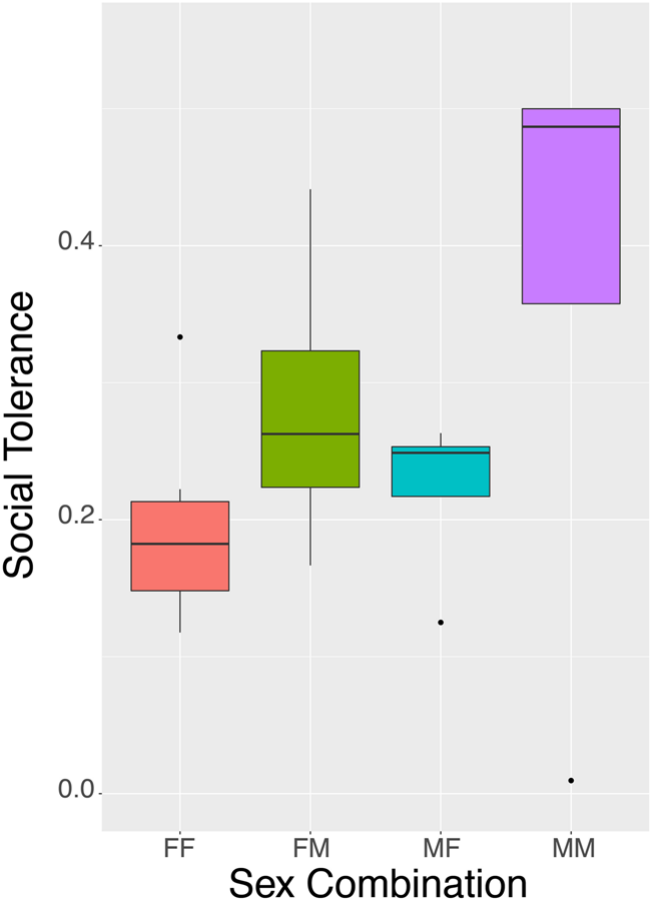
Social Tolerance per combination of the sexes. *FF* female-female, *FM* female-male, *MF* male–female, *MM* male-male. Graph shows median (solid line), 25th and 75th percentile (box) and the largest and smallest value within 1.5 times the interquartile ranges respectively (whiskers).

On average the azure-winged magpies shared mealworms (independent of condition) with 1.9 different partners (range 0 – 4), and actively shared with 1.5 different partners (range 0 – 4). When analyzing the likelihood of sharing with a specific individual, we encountered a problem of multi-collinearity regarding Sex-combination and Social Tolerance, and indeed a GLMM on Social Tolerance (with Subject nested in Group as random effect) showed a clear effect of Sex-Combination on Social Tolerance, with specifically male-male dyads showing high Social Tolerance in comparison to male–female and female-female dyads (MM vs. MF: Estimate = 0.16, t = 3.413, *p* = 0.004; MM vs. FF: Estimate = 0.16, z = 3.357, *p* = 0.007; Fig. 3).

**Figure 4:**
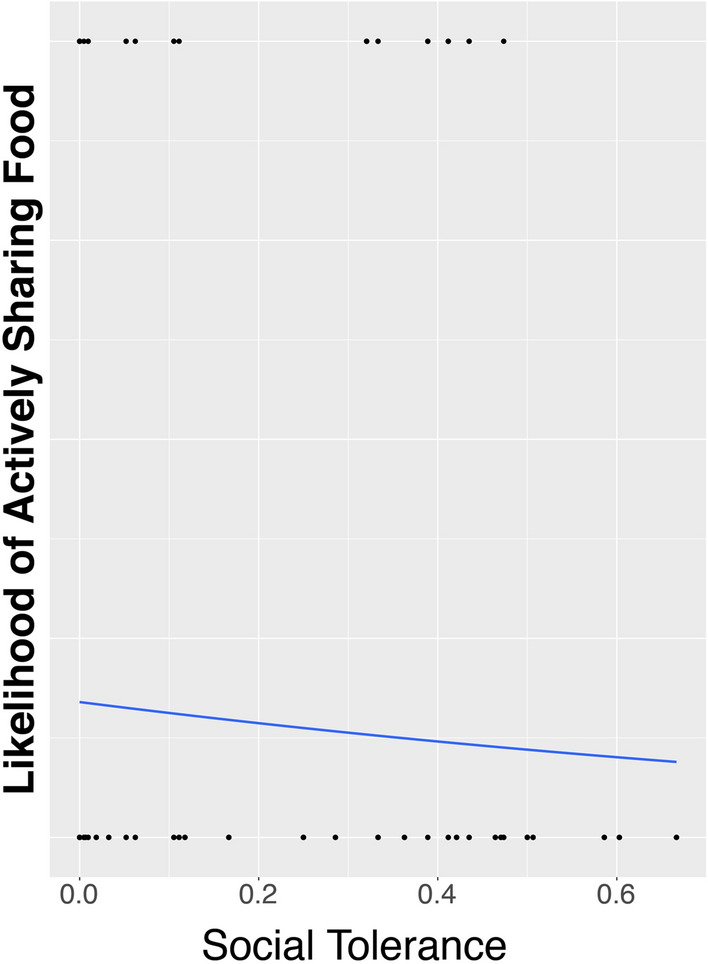
Likelihood of active food sharing in relation to social tolerance. Solid line represents logistic regression line.

**Figure 5:**
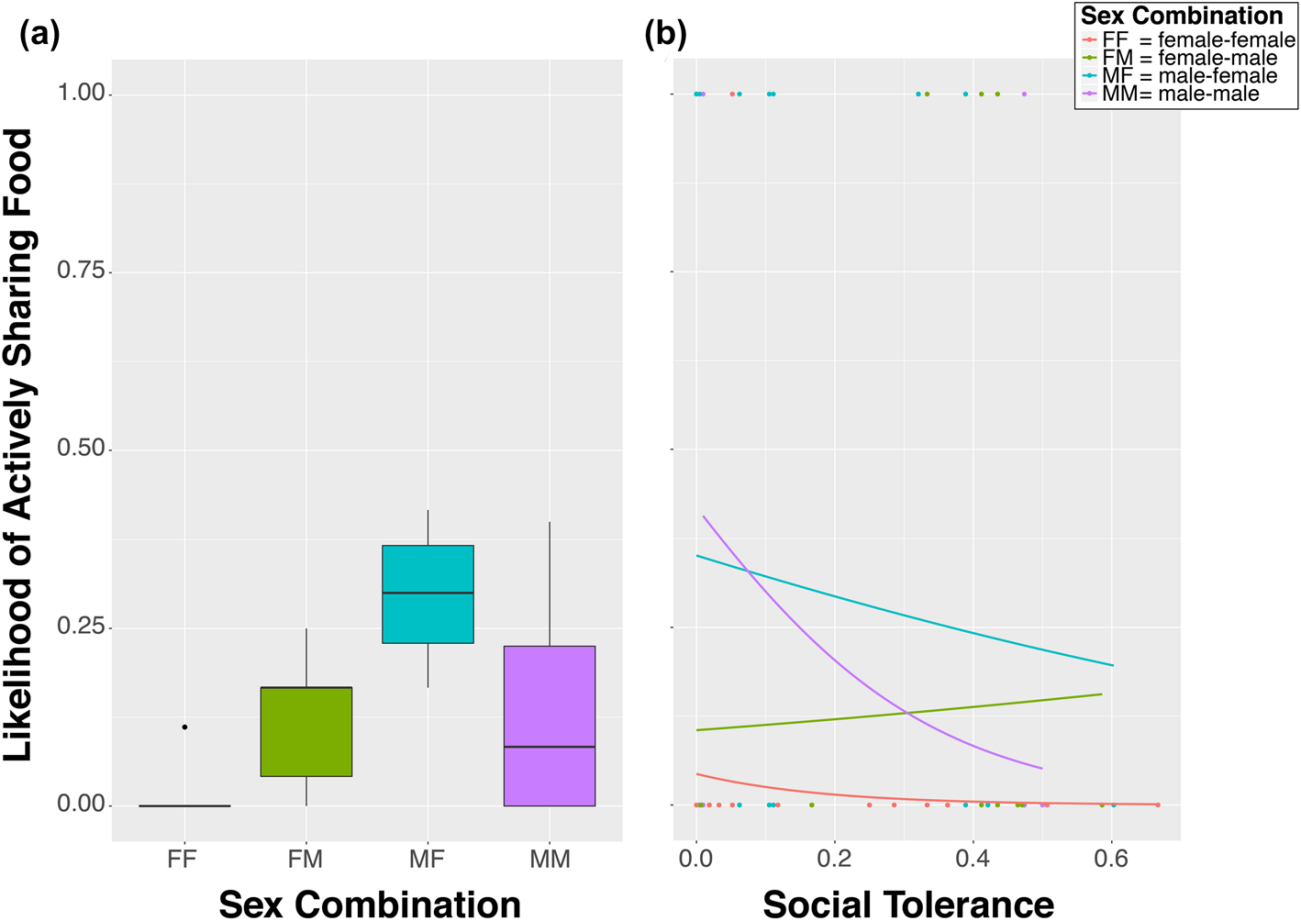
Likelihood of actively sharing mealworms, (**a**) per sex combination, and (**b**) the effect of social tolerance on the likelihood of sharing per combination of the sexes. Boxplots (**a**) show median (solid line), 25th and 75th percentile (box) and the largest and smallest value within 1.5 times the interquartile ranges respectively (whiskers). Solid lines (**b**) represent logistic regression lines.

Therefore, we decided to run three separate models on the likelihood of sharing with a specific individual, one including only Social Tolerance, one with only Sex Combination, and one including only the interaction between Sex Combination and Social Tolerance (all three with Individual nested in group as random effects, and because of its strong effects in the previous analyses, Condition as a control variable (which was thus also included in the null model)). In the first model we found that our measure of Social Tolerance had a significantly negative effect on the likelihood of active sharing with a specific individual (Estimate -1.77, z = -6.298, *p* < 0.001; Fig. 4). In the second model we found a strong effect of sex-combination, showing that both sexes were much more likely to actively share with someone of the opposite sex than with their own sex (FM vs FF: Estimate = 1.72, z = 7.977, *p* < 0.001; MF vs MM: Estimate = 1.04, z = 5.829, *p* < 0.001; Fig. 5a). Finally, in the third model we found an interaction effect between Sex Combination and Social Tolerance, where social tolerance has a positive effect on the likelihood of a female sharing actively with a male, albeit not significantly (Estimate = 0.43, z = 0.842, *p* = 0.40), whereas for all other sex combinations this relationship is significantly negative (MM: Estimate = -4.82, z = -7.873, *p* < 0.001; MF: Estimate = -1.75, z = -4.442, *p* < 0.001; FF: Estimate = -11.91, z = -4.034, *p* < 0.001)(Fig. 5b).

### Vocalizations/requests

**Figure 6:**
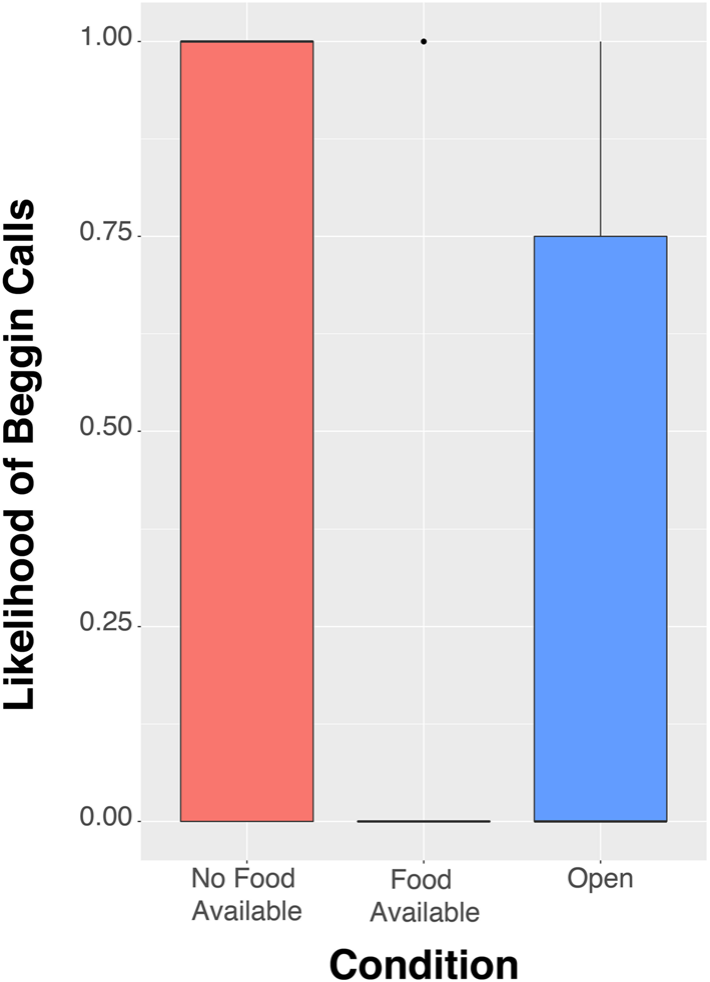
Likelihood of begging calls by the group (excluding the subject bird) when the subject bird had mealworms whereas they themselves did not have access to mealworms (No Food Available), when they also had access to mealworms (Food Available) or in a situation in which all birds had similar access to all compartments as well as to mealworms. Graph shows median (solid line), 25th and 75th percentile (box) and the largest and smallest value within 1.5 times the interquartile ranges respectively (whiskers).

Overall we recorded a total of 147 begging calls from the potential recipients. The birds were, however, far more likely to beg in the No Food Available Condition than in the Food Available (Estimate = 3.91, z = 10.871, *p* < 0.001) or Open Condition (Estimate = 2.52, z = 9.007, *p* < 0.001)(Fig. 6). This contrasted with other call types that did not show any difference in likelihood across conditions (see Supplementary Information). The actual number of begging calls in those sessions in which begging happened (second hurdle) showed a similar but non-significant pattern and the full model did not differ significantly from the null model (see Supplementary Information).

**Figure 7:**
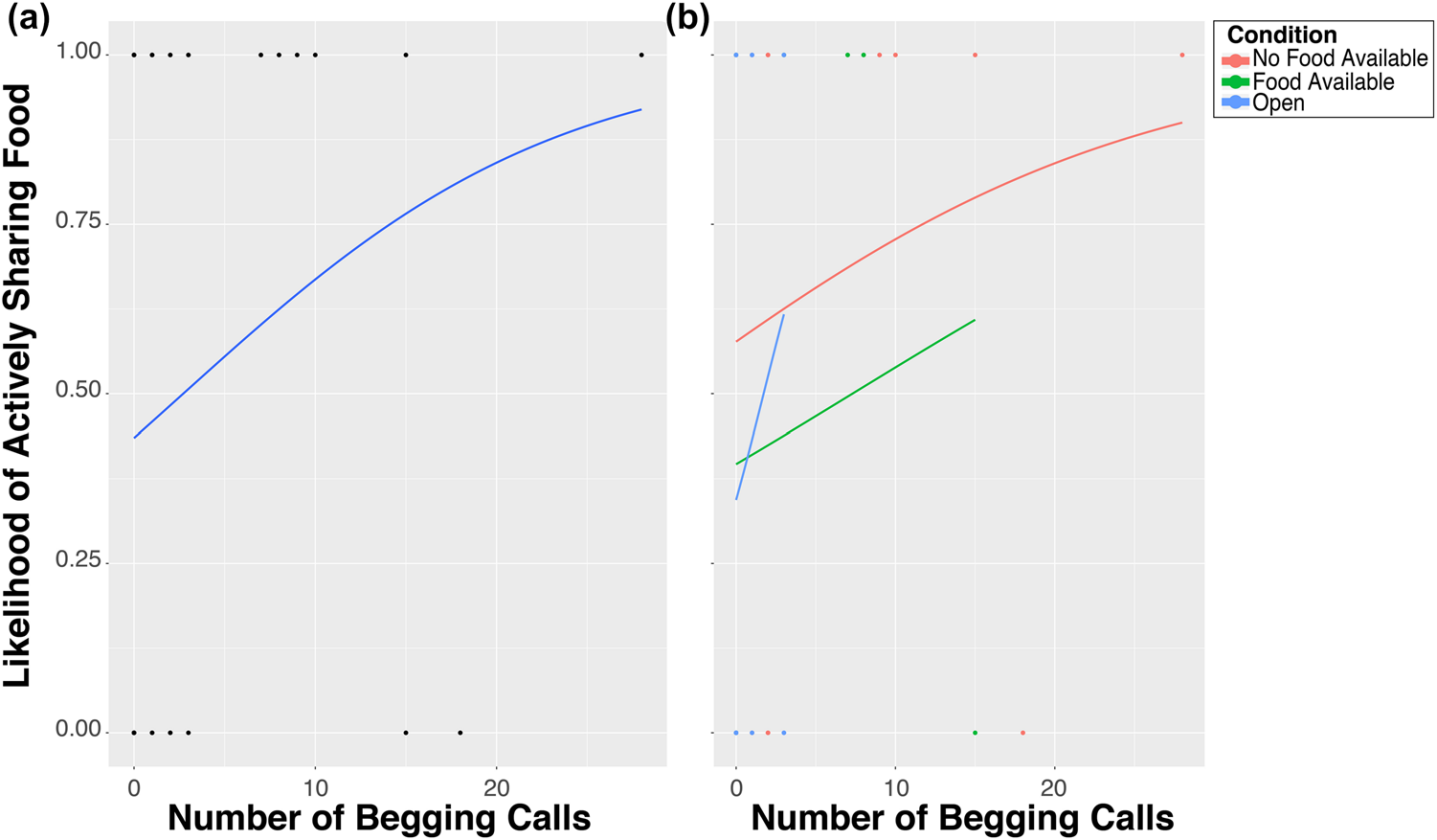
(**a**) Likelihood of active food sharing in relation to the number of begging calls of the potential recipients, and (**b**) split up per condition. Solid lines represent logistic regression lines.

Begging had a significantly positive effect on the likelihood of active sharing (Estimate: 0.07, z = 2.575, *p* = 0.010; Fig. 7a). However, we also found an interaction effect between begging and condition, suggesting that the likelihood of active sharing increased more due to begging in the No Food Available Condition compared to begging in the Food Available Condition (Estimate = 0.883, z.ratio = 2.886, *p* = 0.011; Fig. 7b), and compared to begging in the Open Condition (Estimate = 1.651, z.ratio = 3.055, *p* = 0.006; Fig. 7b). Models on the actual number of active food-sharing in those sessions in which active food-sharing happened (second hurdle) did not differ significantly from the null model (see Supplementary Information). Moreover, when we restricted our original analyses on active sharing to those sessions where there was no begging from the potential recipients we found exactly the same pattern as in the original analyses; i.e. we found a strong effect of condition, namely that the birds were significantly more likely to actively share food in the No Food Available Condition compared to the Food Available Condition (Estimate = 17.50, z = 2.429, *p* = 0.015), as well as when compared to the Open Condition (Estimate = 23.04, z = 2.778, *p* = 0.005)(Figure S1a). Additionally, we found a significant interaction between condition and sex, which showed that it was particularly the females that showed a reduced likelihood to share in the Food Available and Open Condition, as they were less likely to share in these conditions than males were (Food Available, F-M: Estimate = -17.44, z = -1.957, *p* = 0.050; Open, F-M: estimate = -23.771, z = -2.395, *p* = 0.017), whereas there was no difference with regard to their sharing likelihood in the No Food Available condition (Estimate = 0.069, z = 0.009, *p* = 0.993)(Figure S1b), further corroborating the suggestion that only females considered the food availability of their conspecifics when actively sharing food.

## Discussion

Our results show that azure-winged magpies share food regularly with each other, and are more likely to do so when others have none. Specifically females seem to differentiate (as shown by number of occurrences of active sharing) between when their conspecifics have no food versus when they have access to food,whereas males seem to share food with a high frequency regardless of the context their conspecifics are in. Furthermore, the azure-winged magpies shared with more than one partner. Partners of the opposite sex are, however, more likely to receive food than those of the same sex. Social tolerance between the bird with the food and the potential recipients seemed to have a negative effect on the likelihood of sharing. This negative effect was particularly clear for males, whereas for females social tolerance, at least with males, seemed to have a positive effect on the likelihood of sharing. The potential recipients actively begged for the food, and were more likely to beg when they themselves had none. The frequency of begging of those potential recipients had a positive effect on the actor’s likelihood of sharing, although this response seemed dependent on the condition the birds were in and largely restricted to when potential recipients were lacking in food. Moreover, further analyses on only those sessions in which the potential recipients did not beg revealed the same pattern as the original analyses, suggesting that when there is no begging actors can differentiate their food-sharing behavior based on the presence or lack of food for the potential recipients.

Our results on experimentally induced food-sharing in captivity corroborate observations of food-sharing between adult azure-winged magpies in the wild^55–57^. Moreover, our findings support an earlier experimental account of proactive prosociality in azure-winged magpies^12^, yet in a slightly more ecologically relevant setting. Food-sharing among (sub)adult individuals has now been reported for several primate (reviewed in^25^) and bird species, and in the latter most notably among corvids^28–32,34^, although this may represent a sampling bias. Among corvids, food-sharing is not necessarily restricted to just one (pair)-partner. It is suggested to aid in the development of pair-bonds in pre-adulthood^29,32,34^. Nevertheless, there are also reports of food-sharing among same-sex partners and between individuals of opposite sexes, yet outside their existing pair-bond, which has been attributed to costly signaling of dominance^30^, and the initiation of extra-pair copulations or new pair bonds^28^, respectively. Although food-sharing among the azure-winged magpies in this study was not restricted to just one (pair) partner, they did seem to be relatively specific with regard to with whom they share. Both sexes seemed to prefer sharing their food with the opposite sex, suggesting that it may be a behavior that facilitates either the maintenance or the formation of a pair-bond. Although our study population consisted of only one mated breeding pair, pair-bond-like affiliation patterns could be observed among the non-breeding birds (pers. observation). As our population contained only one breeding pair, however, there was not enough power to analyze whether there was an additive effect of being a mated breeding pair on food-sharing. Notwithstanding, we did also witness sharing between members of the same sex, although among males only, as females hardly ever shared with each other (cf.^66^). The fact that males share food among each other may function to reinforce cooperative bonds in this cooperatively breeding species, much like what has recently been reported for both golden-headed lion tamarins, *Leontopithecus chrysomelas*, and red-handed tamarins, *Saigninus midas*^67^. Moreover, this finding corroborates results in another corvid, ravens, *Covus corax*, that show that male-male relationships among non-breeders are much stronger than female-female relationships^68^.

Social tolerance between two individuals seemed to have a negative effect on the likelihood of sharing, although this effect seemed largely driven by males. In contrast, the amount of social tolerance females experience with/from a male did seem to have a positive effect on the likelihood of sharing. These opposing patterns seem to reflect a different function of food-sharing between females and males; i.e. for females food-sharing with those males they already have a good relationship (high social tolerance) with might function as pair-bond maintenance, whereas for males the sharing with females they did not (yet) have a good relationship with may function to establish (extra-) pair bond formation. Males may either not be interested in maintaining already existing social bonds with females, or they use different behaviors, like allopreening^69,70^, to do so. The effect of social tolerance on the likelihood of same-sex sharing remains difficult to interpret since first, females did almost never share with each other, and second, because each male had only one other male in its group to potentially share with.

The female azure-winged magpies in this study, moreover, were more likely to share mealworms with their conspecifics when these others had no access to mealworms than when they also had access to mealworms. This suggests that they differentiate between when their conspecifics have food or do not have food.. These findings corroborate findings on another corvid, Eurasian jays^31^ and yet another bird species, New Zealand robins^33^, and are at par with findings on chimpanzees^43^ and capuchin monkeys^71^. The studies on the Eurasian jays, New Zealand Robins and chimpanzees, however, have also been shown to flexibly adjust their help/sharing based somehow on the current desire of their conspecific^31,33,43^. Whether azure-winged magpies can do so too requires further testing.

In contrast to the females, the males shared their mealworms regardless of whether others also had access to mealworms. Again this suggests that the function of food-sharing in male and female azure-winged magpies may differ; i.e. whereas females seem to take into account whether or not their conspecifics have access to food, males are always generous, possibly to attract new mates (see above), or to signal their status (cf.^72^; see also^30^). An investigation into the more proximate mechanisms leading to food-sharing may shed light on these patterns. For example, Duque and colleagues found that administering mesotocin, the avian analogue of mammalian oxytocin, increased food-sharing in a prosocial choice task in pinyon jays^11^, and responses to oxytocin, and also vasopressin are notoriously different between the sexes^73^.

From a behavioral standpoint, we show that the azure-winged magpies reacted to the begging of their conspecifics. The group members begged more in the condition in which they had no access to mealworms, and the subject birds were more likely to share their mealworms when their conspecifics were begging. This finding is similar to findings in chimpanzees that also showed that those that don’t have access to a specific food item beg frantically and those that have the food item seem to react with sharing^24,43,45^, although there are also reports that show negative effects of requests on prosocial sharing in both chimpanzees and orangutans in a food-sharing task^47^ and of attention-getting behavior in chimpanzees^46^.

From a cognitive perspective, this begging could be interpreted as a cue, and the subsequent sharing as only a reaction to this cue. Consequently, the food-sharing would be of zero-order intentionality only^52^. However, we did also witness sharing without begging, and when we analyzed only those sessions in which we did not observe any begging, we found exactly the same patterns; i.e. azure-winged magpies, and particularly the females, are more likely to share when their group members do not have access to the desired food, than when they do (figure S1). Moreover, simple responses to a begging cue would not explain the sex differences we found, though there might be other cues that accompany the begging calls that we did not consider. Additionally, we found an interaction effect between begging and condition on the likelihood of sharing, suggesting that whether the birds respond to begging still depends on the availability of food of the begging birds; i.e. this response seems larger when the begging conspecifics actually lack food. Nevertheless, caution is needed in the interpretation of this interaction effect, as there was in general less begging as well as less sharing in the control conditions in comparison to the test (No Food Available) condition. Consequently, to truly tap into the levels of intentionality of azure-winged magpie food-sharing, and whether it involves some sort of desire-state attribution, further testing is necessary.

In sum, adult azure-winged magpies show proactive food-sharing among conspecifics. Although the majority of food-sharing events are between the sexes, suggestive of pair bond formation and/or maintenance, they are not exclusive to those bonds. Most notably, the azure-winged magpies, and specifically the females of this species, pay attention to the food availability of their conspecifics and subsequently cater to this . Begging for food may function as a cue eliciting this food-sharing, although depending on the availability of food to their conspecifics, the azure-winged magpies also shared food when their conspecifics did not beg, and further tests are required to elucidate the cognitive underpinnings of food-sharing in azure-winged magpies.

## Supplementary information

Supplementary Information.

## Data Availability

Raw data are made available on an online repository; i.e. DataverseNL. Link: 10.34894/TGPGBE
