# Supplementary material for: Azure-winged magpies’ decisions to share food are contingent on the presence or absence of food for the recipient
Source: Sci Rep. 2020 Sep 30;10:16147. doi: 10.1038/s41598-020-73256-0 (PMC7528063; doi:10.1038/s41598-020-73256-0)
Supplement: Supplementary file 1 — Supplementary Information. [file 41598_2020_73256_MOESM1_ESM.docx]

**Supplemental Information for:**

**Massen, J.J.M.^*^,** Haley, S.M.^*^ & Bugnyar, T. (*in review*). Azure-winged magpies’ decisions to share food are contingent on the presence or absence of food for the recipient

**Index:**

**Ethogram 2**

**Figure S1 4**

**Results on Total Sharing 5**

**Model Outputs 10**

**Caching 10**

**Total Sharing**

**(TS; i.e. both active and passive sharing) 11**

**Active Sharing (AFS) 13**

**Social Tolerance 15**

**Total Sharing (Y/N)**

**per dyad including Social Tolerance 16**

**Total Sharing (Y/N)**

**per dyad including Sex Combination 17**

**Total Sharing (Y/N) per dyad including Sex Combination*Social Tolerance 19**

**Active Sharing (Y/N) per dyad including Social Tolerance** **20**

**Active Sharing (Y/N) per dyad including Sex Combination** **21**

**Active Sharing (Y/N) per dyad including Sex Combination*Social Tolerance 23**

**Begging 24**

**Total Sharing including Begging 26**

**Active Sharing including Begging 29**

**Additional Analyses on other call types 32**

**R-Script 34**

**Ethogram for Focal Individual / Group:**

Vocalizations:

**Contact**- relatively short and projecting calls with ascending pitch and not obviously directed towards anyone in particular, either in response to or with the response of other contact calls

**Begging**- soft, high-pitched peeping directed towards individual that has food. Often accompanied by following behavior; i.e. closely (within 10 cm proximity) following the other individual via hopping on the ground or in tree branches ; and wing fluttering

**Soft Calls**- soft/quiet calls with range of pitches and durations (including peeping) that are usually not directed towards anyone in particular, but can be in response to other calls

**Chattering**- vocalizations with a lot of variation (of pitch, duration, articulation, call type, etc.) going back and forth between 2 or more individuals; can also occur with just one individual in rare cases. Record all individuals involved.

**Excitement**- series of short, loud calls made when not in observable danger or threat, usually in response to food

**Distress**- series of short, loud calls made when threatened

**Threat**- harsh, loud, and noisy one-syllable calls directed towards a particular individual (bird or human)

**Mating Br**- including several characteristic elements: drawn out, sliding syllables that usually end with a rattle and sometimes begin with a peep. Number of syllables varies and calls often contain shorter sliding syllables right before the rattle. Only performed by females. Indicate who makes the vocalization, and when possible, to whom.

**Mating P**- high-pitched squeaking right before and during intercourse; can be comprised of sliding syllables or short syllables more similar to those used when chattering. Indicate the two individuals participating.

**Alarm**- loud, sharp, and harsh series of syllables in response to a threat to the group; usually more than one individual participates at a time, often synchronized.

**Other**- can’t be classified in any of the above categories

Sharing:

**Active share**- gives mealworms to another individual through beak-to-beak contact

**Passive share**- lets another bird take food without actively offering or protesting

**REC food**- is given food (actively or passively) by another bird

**Visit border with food**- flies/rests within 10cm of the border while visibly in possession of mealworms

**Visit border no food**- flies/rests within 10 cm of the border while not visibly in possession of mealworms

Caching:

**Caching A**- purposefully leaves or hides food at any place in the cage not at the border

**Caching B**- purposefully hides food in the ground at the border

**Leaving food at the border**- purposefully leaves food at the border in a position where birds on the other side of the cage could potentially reach it

Aggressive behaviors:

**Displacement**- removes another bird from its position simply by arriving at the same location.

**REC Displacement**- leaves resting area when another bird arrives.

**Pecking**- sharply and aggressively hits another bird with beak.

**REC Pecking**- another bird sharply and aggressively hits focal with beak.

**Chasing**- aggressively follows another bird around the cage, displacing it.

**REC Chasing**- another bird aggressively follows focal around the cage, displacing it.

**Fight**- two birds engage in aggressive behavior towards each other

**Stealing**- one bird forcefully takes worms from another bird.

**REC Stealing**- another bird forcefully takes worms from the focal.

Neutral/positive behaviors:

**Scratch**- uses its foot to itch an area of its body

**Auto-preening**- runs its beak over its feathers

**Allo-preening**- runs its beak over another bird’s feathers.

**REC Allo-preening**- another bird runs its beak over focal bird’s feathers.

**Yawning**- opens its mouth for a short but significant duration without vocalizing or expelling food

**Bill twining**- two birds touch/hold beaks without sharing food.

**Following**- follows another bird in a non-aggressive way, usually begging for food

**REC following**- another bird follows focal in a non-aggressive manner


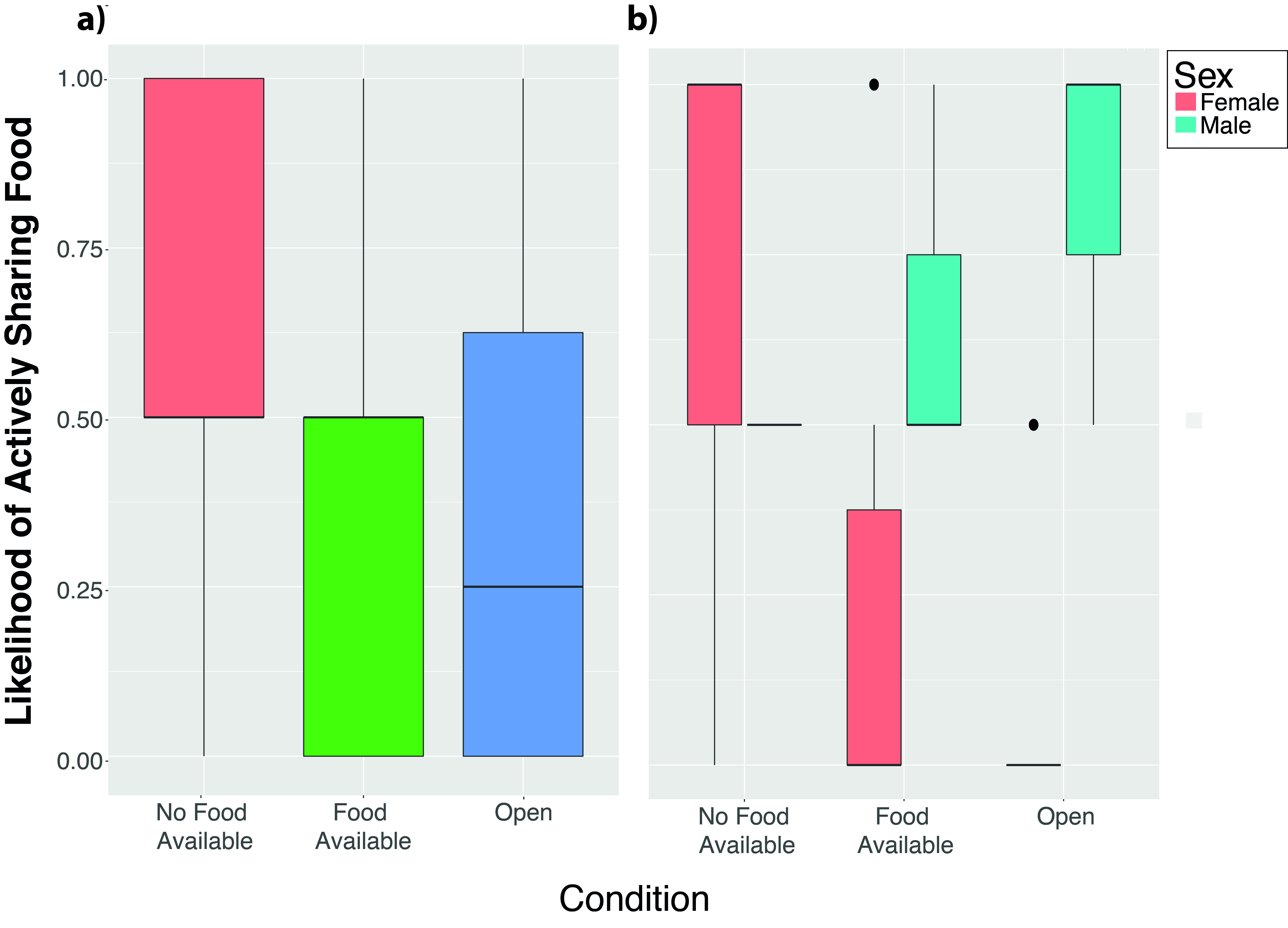


**Figure S1: Likelihood of actively sharing mealworms by the subjects, excluding those sessions in which there was begging of potential recipients, a)** when their conspecifics did not have access to mealworms (No Food Available), when they also had access to mealworms (Food Available) or in a situations in which all birds had similar access to all compartments as well as to mealworms, and **b) split up for the different sexes.** Graphs show median (solid line), 25th and 75th percentile (box) and the largest and smallest value within 1.5 times the interquartile ranges respectively (whiskers).

**Results on Total Sharing**

*Sharing*

When analyzing the likelihood of sharing, we found a strong effect of condition, which showed that the birds were more likely to share food in the No Food Available Condition compared to the Food Available Condition (Estimate = - 1.56, z = -5.317, P < 0.001), as well as when compared to the Open Condition (Estimate = - 2.71, z = -8.489, P < 0.001)(Figure S1a). Additionally we found an interaction between condition and sex, which showed that whereas females seemed to differentiate between the conditions (No Food vs Food Available: Estimate = 1.56, z = 5.317, P < 0.001; No Food Available vs. Open: Estimate = 2.71, z = 8.489, P < 0.001; No Need vs. Open: estimate = 1.14, z = 4.024; figure S1b), males did not show any significant differences between the conditions, suggesting that only females considered the need of their conspecifics when sharing food. When considering the number of food-sharing events in those sessions in which food-sharing happened (second hurdle), we found similar patterns (see model outputs), albeit not significantly, and moreover, this model did not differ from a null model containing only random effects on the same data.

**
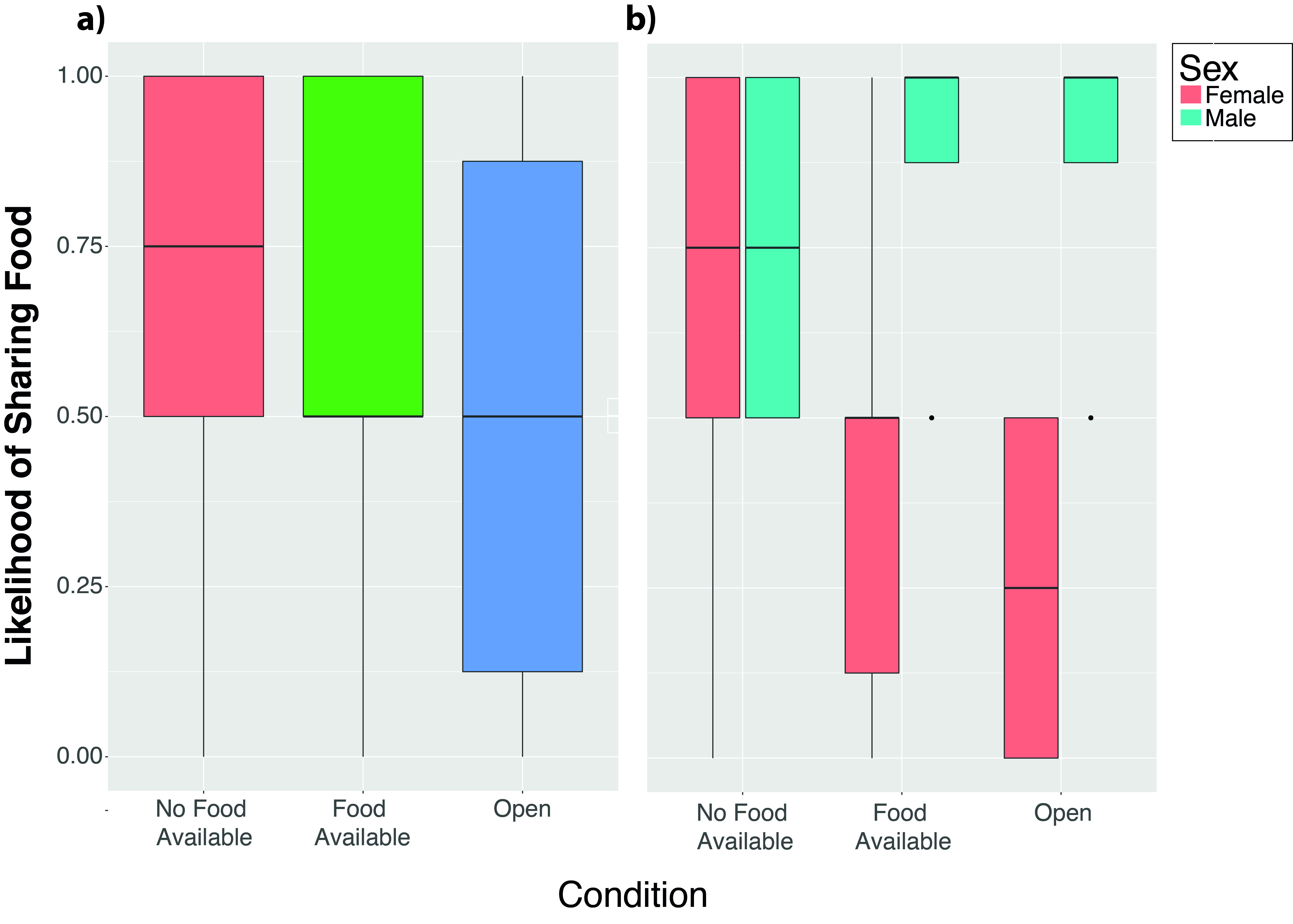
Figure S2: Likelihood of sharing** mealworms by the subjects **a)** when their conspecifics did not have access to mealworms (No Food Available), when they also had access to mealworms (Food Available) or in a situations in which all birds had similar access to all compartments as well as to mealworms, and **b) split up for the different sexes.** Graphs show median (solid line), 25th and 75th percentile (box) and the largest and smallest value within 1.5 times the interquartile ranges respectively (whiskers).

*Sharing with whom*

In the first (see main text for rationale of the three separate models) model we found that our measure of Social Tolerance had a significantly negative effect on the likelihood of sharing with a specific individual (Estimate -1.25, z = -4.812, p < 0.001), albeit not very strong (see Figure S2).

In the second model we found a strong effect of sex-combination, showing that both sexes were much more likely to share with someone of the opposite sex than with their own sex (FM vs FF: Estimate = 0.59, z = 3.992, p <0.001; MF vs MM: Estimate = 1.24, z = 6.960, p <0.001; Figure S3a). Finally, in the third model we found an interaction effect between Sex Combination and Social Tolerance, where social tolerance has a significantly positive effect on the likelihood of a female sharing with a male (Estimate = 1.08, z = 2.482, p = 0.013), whereas for males sharing with both males and females this relationship is significantly negative (MM: Estimate = -5.37, z = -8.735, p < 0.001; MF: Estimate = -1.62, z = -4.193, p < 0.001)(Figure S3b).

**
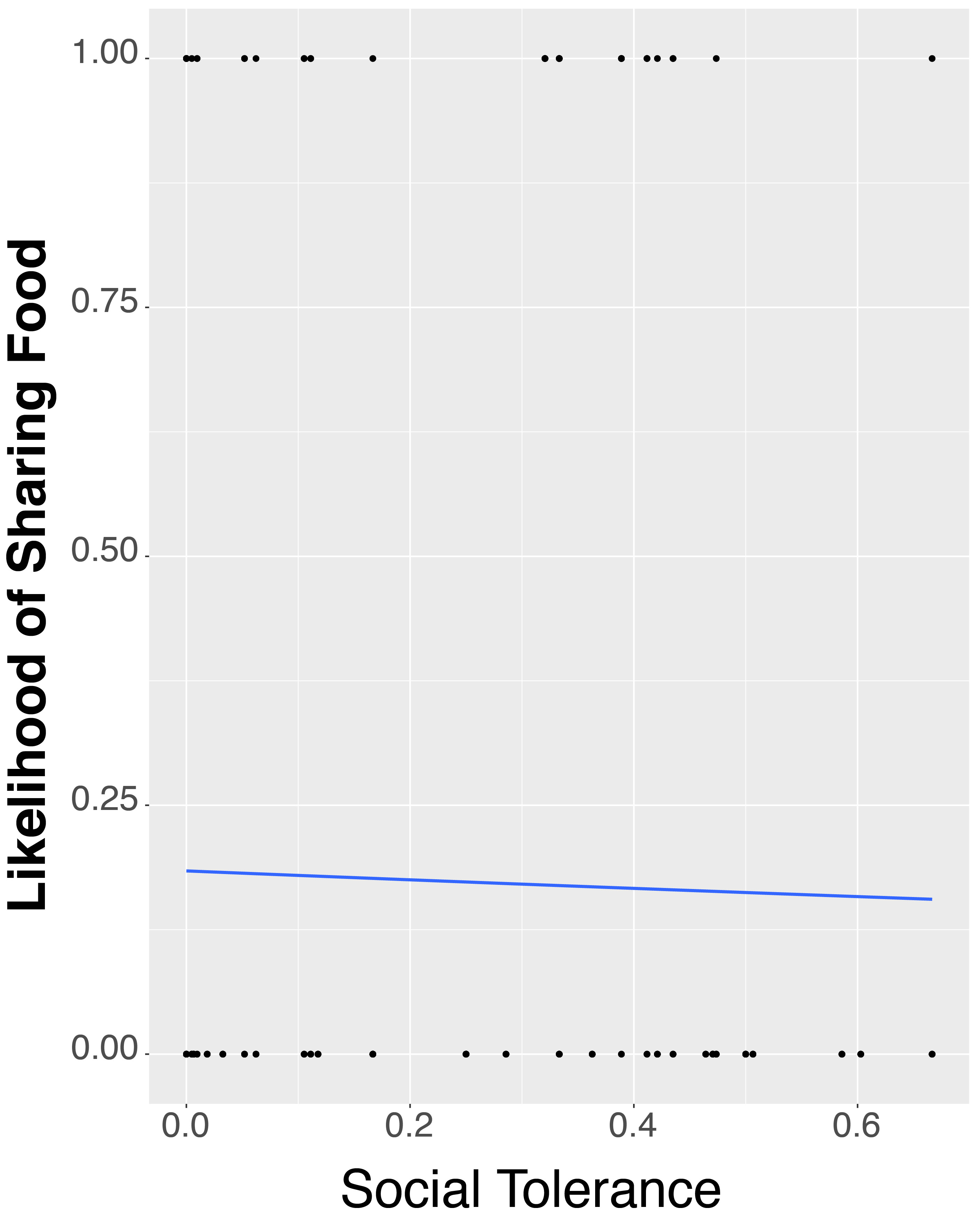
**

**Figure S3: Likelihood of total food sharing in relation to social tolerance.** Solid line represents logistic regression line.

**
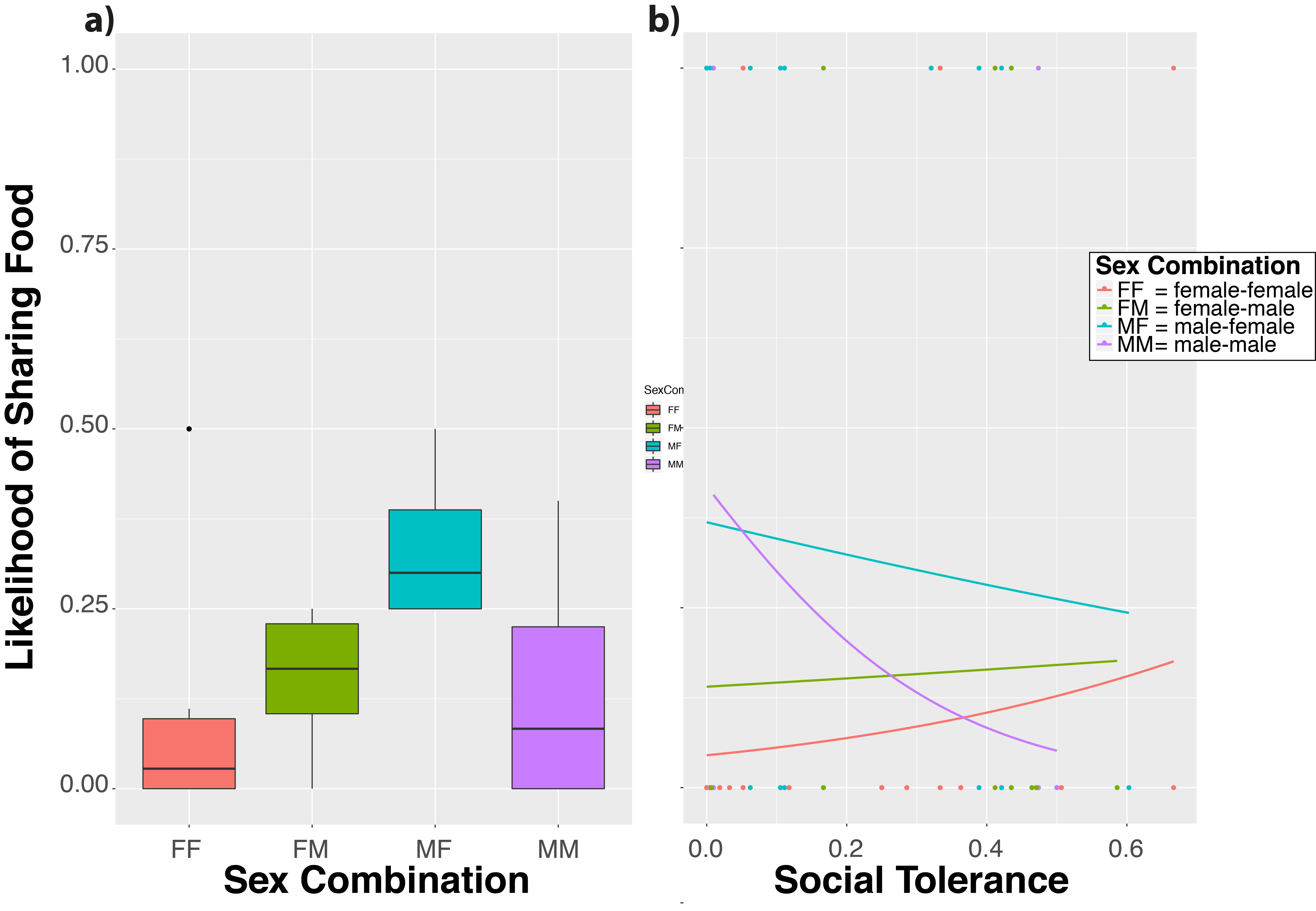
Figure S4: Likelihood of sharing** mealworms, **a) per sex combination,** and **b) the effect of social tolerance on the likelihood of sharing** per combination of the sexes**.** Boxplots (a) show median (solid line), 25th and 75th percentile (box) and the largest and smallest value within 1.5 times the interquartile ranges respectively (whiskers). Solid lines (b) represent logistic regression lines.

*Vocalizations / requests*

Begging had a significantly positive effect on the likelihood of sharing (Estimate: 0.06, z = 2.395, p = 0.017; figure S4a). But, we also found an interaction effect between begging and condition, suggesting that the likelihood of total sharing increased more due to begging in the No Food Available condition compared to the Food Available condition (total sharing: Estimate = 0.836, z.ratio = 2.893, p = 0.011; figure S4b), but not compared to the open condition (Estimate = -0.426, z.ratio = -0.803, p = 0.701; figure S4b). Models on the actual number of food sharing in those sessions in which food sharing happened (second hurdle) did not differ significantly from the null model (see model outputs).

**
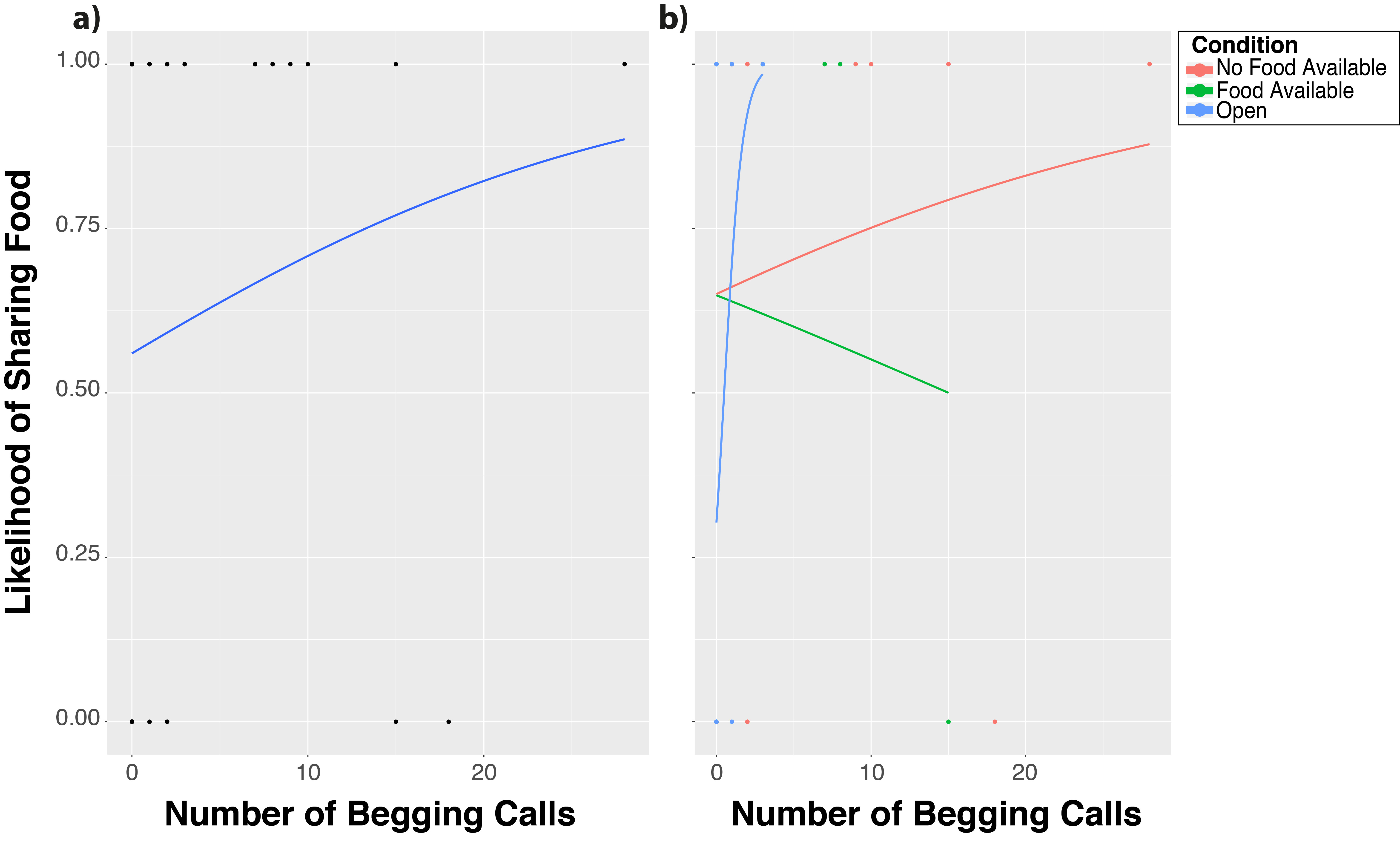
Figure S5: a) Likelihood of total food sharing in relation to the number of begging calls** of the potential recipients, and **b) split up per condition.** Solid lines represent logistic regression lines.

**Model Outputs**

***#Note that Condition A is the Need Condition, Condition B the No Need Condition, and Condition C the Open Condition#***

**Caching**

CACHEnull: CACHE ~ (1 | Group/Individual)

CACHE: CACHE ~ Condition + Sex + Condition * Sex + (1 | Group/Individual)

Df AIC BIC logLik deviance Chisq Chi Df

CASHEnull 4 657.51 665.75 -324.75 649.51

CASHE 9 662.50 681.05 -322.25 644.50 5.0059 5

Pr(>Chisq)

CASHEnull

CASHE 0.4152

***Formula: CACHE ~ Condition + Sex + Condition * Sex + (1 | Group/Individual)***

Weights: Weight

REML criterion at convergence: 595.2

Scaled residuals:

Min 1Q Median 3Q Max

-1.5376 -0.5622 -0.2347 0.1815 3.2810

Random effects:

Groups Name Variance Std.Dev.

Individual:Group (Intercept) 741.2 27.23

Group (Intercept) 1143.8 33.82

Residual 53284.9 230.84

Number of obs: 58, groups: Individual:Group, 10; Group, 2

Fixed effects:

Estimate Std. Error df t value Pr(>|t|)

(Intercept) 61.248 31.692 1.935 1.933 0.1973

ConditionB -36.346 25.270 44.146 -1.438 0.1574

ConditionC -43.029 24.548 43.861 -1.753 0.0866

SexM -2.548 32.585 20.840 -0.078 0.9384

ConditionB:SexM 14.821 39.072 43.957 0.379 0.7063

ConditionC:SexM 10.007 39.497 44.096 0.253 0.8012

**Total Sharing (TS; i.e. both active and passive sharing)**

***First Hurdle: Likelihood***

Sbinnull: TSBIN ~ (1 | Group/Individual)

TSbinom: TSBIN ~ Condition + Sex + Condition * Sex + (1 | Group/Individual)

Df AIC BIC logLik deviance Chisq Chi Df

TSbinnull 3 825.27 831.45 -409.63 819.27

TSbinom 8 727.82 744.31 -355.91 711.82 107.45 5

Pr(>Chisq)

TSbinnull

TSbinom < 2.2e-16 ***

***Formula: TSBIN ~ Condition + Sex + Condition * Sex + (1 | Group/Individual)***

Weights: Weight

AIC BIC logLik deviance df.resid

727.8 744.3 -355.9 711.8 50

Scaled residuals:

Min 1Q Median 3Q Max

-7.9525 -1.4135 0.1713 2.2143 7.3006

Random effects:

Groups Name Variance Std.Dev.

Individual:Group (Intercept) 8.043e+00 2.836e+00

Group (Intercept) 7.869e-09 8.871e-05

Number of obs: 58, groups: Individual:Group, 10; Group, 2

Fixed effects:

Estimate Std. Error z value Pr(>|z|)

(Intercept) 0.6289 1.2063 0.521 0.602

ConditionB -1.5642 0.2942 -5.317 1.06e-07 ***

ConditionC -2.7068 0.3189 -8.489 < 2e-16 ***

SexM 2.4101 2.1231 1.135 0.256

ConditionB:SexM 2.6588 0.4919 5.405 6.48e-08 ***

ConditionC:SexM 3.7977 0.5076 7.482 7.31e-14 ***

***Second Hurdle: Number of sharing events when food-sharing occurred***

TSnull: TS ~ (1 | Group/Individual)

TS: TS ~ Condition + Sex + Sex * Condition + (1 | Group/Individual)

Df AIC BIC logLik deviance Chisq Chi Df

TSnull 4 -2318 -2312 1163 -2326

TS 9 1106090 1106104 -553036 1106072 0 5

Pr(>Chisq)

TSnull

TS 1

***Formula: TS ~ Condition + Sex + Sex * Condition + (1 | Group/Individual)***

Data: subset(xdata, TS > 0)

Weights: Weight

AIC BIC logLik deviance df.resid

1106090 1106104 -553036 1106072 26

Scaled residuals:

Min 1Q Median 3Q Max

4.355 10.000 17.590 29.614 90.385

Random effects:

Groups Name Variance Std.Dev.

Individual:Group (Intercept) 0 0

Group (Intercept) 0 0

Number of obs: 35, groups: Individual:Group, 9; Group, 2

Fixed effects:

Estimate Std. Error z value Pr(>|z|)

(Intercept) -1.0731 0.1542 -6.959 3.44e-12 ***

ConditionB -1.3047 0.4063 -3.211 0.00132 **

ConditionC -0.7981 0.4081 -1.955 0.05052 .

SexM -0.3520 0.2626 -1.340 0.18015

ConditionB:SexM 0.8382 0.5211 1.609 0.10769

ConditionC:SexM 0.4202 0.5274 0.797 0.42557

**Active Sharing (AFS)**

***First Hurdle: Likelihood***

AFSbinnull: FASBIN ~ (1 | Group/Individual)

AFSbinom: FASBIN ~ Condition + Sex + Condition * Sex + (1 | Group/Individual)

Df AIC BIC logLik deviance Chisq Chi Df

AFSbinnull 3 849.19 855.38 -421.60 843.19

AFSbinom 8 728.87 745.35 -356.43 712.87 130.33 5

Pr(>Chisq)

AFSbinnull

AFSbinom < 2.2e-16 ***

***Formula: FASBIN ~ Condition + Sex + Condition * Sex + (1 | Group/Individual)***

Weights: Weight

AIC BIC logLik deviance df.resid

728.9 745.4 -356.4 712.9 50

Scaled residuals:

Min 1Q Median 3Q Max

-9.2091 -1.7163 0.0033 1.7680 9.1586

Random effects:

Groups Name Variance Std.Dev.

Individual:Group (Intercept) 6.854e+00 2.618e+00

Group (Intercept) 2.077e-09 4.558e-05

Number of obs: 58, groups: Individual:Group, 10; Group, 2

Fixed effects:

Estimate Std. Error z value Pr(>|z|)

(Intercept) 0.0248 1.1018 0.023 0.982

ConditionB -2.4576 0.3008 -8.170 3.09e-16 ***

ConditionC -2.5057 0.2996 -8.362 < 2e-16 ***

SexM 2.9691 1.8338 1.619 0.105

ConditionB:SexM 2.4576 0.4737 5.189 2.12e-07 ***

ConditionC:SexM 3.6322 0.4994 7.274 3.50e-13 ***

***Second Hurdle: Number of active sharing events when food-sharing occurred***

AFSnull: FAS ~ (1 | Group/Individual)

AFS: FAS ~ Condition + Sex + Sex * Condition + (1 | Group/Individual)

Df AIC BIC logLik deviance Chisq ChiDf

AFSnull 4 181 187 -87 173

AFS 9 2635124 2635136 -1317553 2635106 0 5

Pr(>Chisq)

AFSnull

AFS 1

***Formula: FAS ~ Condition + Sex + Sex * Condition + (1 | Group/Individual)***

Data: subset(xdata, FAS > 0)

Weights: Weight

AIC BIC logLik deviance df.resid

2635124 2635136 -1317553 2635106 20

Scaled residuals:

Min 1Q Median 3Q Max

4.511 8.740 17.984 31.456 85.347

Random effects:

Groups Name Variance Std.Dev.

Individual:Group (Intercept) 1.614e-14 1.270e-07

Group (Intercept) 7.538e-15 8.682e-08

Number of obs: 29, groups: Individual:Group, 9; Group, 2

Fixed effects:

Estimate Std. Error z value Pr(>|z|)

(Intercept) -1.10761 0.16719 -6.625 3.47e-11 ***

ConditionB -1.19768 0.58225 -2.057 0.0397 *

ConditionC -0.50724 0.42990 -1.180 0.2380

SexM -0.40248 0.27555 -1.461 0.1441

ConditionB:SexM 0.91151 0.66752 1.366 0.1721

ConditionC:SexM 0.07855 0.54952 0.143 0.8863

**Social Tolerance**

Type III Analysis of Variance Table with Satterthwaite's method

Sum Sq Mean Sq NumDF DenDF F value Pr(>F)

SEXCombi 0.63269 0.2109 3 237.45 5.6042 0.0009945

$`pairwise differences of SEXCombi`

contrast estimate SE df t.ratio p.value

FF - FM -0.0741 0.0324 237.0 -2.287 0.1038

FF - MF 0.0021 0.0331 14.5 0.064 0.9999

FF - MM -0.1590 0.0474 66.2 -3.357 0.0070

FM - MF 0.0762 0.0332 18.3 2.293 0.1363

FM - MM -0.0850 0.0470 67.8 -1.809 0.2782

MF - MM -0.1611 0.0472 234.5 -3.413 0.0042

P value adjustment: tukey method for comparing a family of 4 estimates

**Total Sharing (Y/N) per dyad including Social Tolerance**

TSperdyadBINnull: TSBIN ~ Condition + (1 | Group/Subject)

TSperdyadBINst: TSBIN ~ Condition + SocTol + (1 | Group/Subject)

Df AIC BIC logLik deviance Chisq Chi Df

TSperdyadBINnull 5 2958.4 2975.9 -1474.2 2948.4

TSperdyadBINst 6 2936.4 2957.4 -1462.2 2924.4 24.002 1

Pr(>Chisq)

TSperdyadBINnull

TSperdyadBINst 9.624e-07 ***

***Formula: TSBIN ~ Condition + SocTol + (1 | Group/Subject)***

Weights: Weight

AIC BIC logLik deviance df.resid

2936.4 2957.4 -1462.2 2924.4 236

Scaled residuals:

Min 1Q Median 3Q Max

-3.7972 -1.9316 -1.2715 -0.2933 16.0069

Random effects:

Groups Name Variance Std.Dev.

Subject:Group (Intercept) 1.9871 1.4097

Group (Intercept) 0.2006 0.4479

Number of obs: 242, groups: Subject:Group, 10; Group, 2

Fixed effects:

Estimate Std. Error z value Pr(>|z|)

(Intercept) -1.1717 0.5786 -2.025 0.042856 *

ConditionB -0.4091 0.1078 -3.794 0.000148 ***

ConditionC -0.8149 0.1197 -6.806 1.0e-11 ***

SocTol -1.2474 0.2592 -4.812 1.5e-06 ***

**Total Sharing (Y/N) per dyad including Sex Combination**

TSperdyadBINnull: TSBIN ~ Condition + (1 | Group/Subject)

TSperdyadBINsc: TSBIN ~ Condition + SEXCombi + (1 | Group/Subject)

Df AIC BIC LogLik deviance Chisq Chi Df

TSperdyadBINnull 5 2958.4 2975.9 -1474.2 2948.4

TSperdyadBINsc 8 2887.5 2915.4 -1435.7 2871.5 76.964 3

Pr(>Chisq)

TSperdyadBINnull

TSperdyadBINsc < 2.2e-16 ***

***Formula: TSBIN ~ Condition + SEXCombi + (1 | Group/Subject)***

Weights: Weight

AIC BIC logLik deviance df.resid

2887.5 2915.4 -1435.7 2871.5 234

Scaled residuals:

Min 1Q Median 3Q Max

-3.9721 -1.8231 -1.1386 -0.3122 14.2476

Random effects:

Groups Name Variance Std.Dev.

Subject:Group (Intercept) 1.22516 1.1069

Group (Intercept) 0.03589 0.1894

Number of obs: 242, groups: Subject:Group, 10; Group, 2

Fixed effects:

Estimate Std. Error z value Pr(>|z|)

(Intercept) -2.3712 0.5473 -4.333 1.47e-05 ***

ConditionB -0.4172 0.1090 -3.828 0.000129 ***

ConditionC -0.8298 0.1209 -6.864 6.69e-12 ***

SEXCombiFM 0.5912 0.1481 3.992 6.55e-05 ***

SEXCombiMF 2.0267 0.7675 2.641 0.008277 **

SEXCombiMM 0.7880 0.7821 1.008 0.313634

$`emmeans of SEXCombi`

SEXCombi emmean SE df asymp.LCL asymp.UCL

FF -2.79 0.545 Inf -3.86 -1.719

FM -2.20 0.540 Inf -3.25 -1.137

MF -0.76 0.574 Inf -1.88 0.365

MM -2.00 0.592 Inf -3.16 -0.838

Results are averaged over the levels of: Condition

Results are given on the logit (not the response) scale.

Confidence level used: 0.95

$`pairwise differences of SEXCombi`

contrast estimate SE df z.ratio p.value

FF - FM -0.591 0.148 Inf -3.992 0.0004

FF - MF -2.027 0.768 Inf -2.641 0.0412

FF - MM -0.788 0.782 Inf -1.008 0.7450

FM - MF -1.436 0.764 Inf -1.879 0.2372

FM - MM -0.197 0.779 Inf -0.253 0.9943

MF - MM 1.239 0.178 Inf 6.960 <.0001

Results are averaged over the levels of: Condition

Results are given on the log odds ratio (not the response) scale.

P value adjustment: tukey method for comparing a family of 4 estimates

**Total Sharing (Y/N) per dyad including Sex Combination*Social Tolerance**

TSperdyadBINnull: TSBIN ~ Condition + (1 | Group/Subject)

TSperdyadBINint: TSBIN ~ Condition + SEXCombi:SocTol + (1 | Group/Subject)

Df AIC BIC logLik deviance Chisq Chi Df

TSperdyadBINnull 5 2958.4 2975.9 -1474.2 2948.4

TSperdyadBINint 9 2841.0 2872.4 -1411.5 2823.0 125.46 4

Pr(>Chisq)

TSperdyadBINnull

TSperdyadBINint < 2.2e-16 ***

***Formula: TSBIN ~ Condition + SEXCombi:SocTol + (1 | Group/Subject)***

Weights: Weight

AIC BIC logLik deviance df.resid

2841.0 2872.4 -1411.5 2823.0 233

Scaled residuals:

Min 1Q Median 3Q Max

-4.8821 -1.8353 -1.1068 -0.2621 13.8614

Random effects:

Groups Name Variance Std.Dev.

Subject:Group (Intercept) 3.0018 1.733

Group (Intercept) 0.1129 0.336

Number of obs: 242, groups: Subject:Group, 10; Group, 2

Fixed effects:

Estimate Std. Error z value Pr(>|z|)

(Intercept) -1.4027 0.6399 -2.192 0.028364 *

ConditionB -0.4260 0.1100 -3.872 0.000108 ***

ConditionC -0.8461 0.1221 -6.929 4.25e-12 ***

SEXCombiFF:SocTol 0.7320 0.4377 1.672 0.094435 .

SEXCombiFM:SocTol 1.0764 0.4338 2.482 0.013077 *

SEXCombiMF:SocTol -1.6198 0.3863 -4.193 2.75e-05 ***

SEXCombiMM:SocTol -5.3699 0.6148 -8.735 < 2e-16 *****Active Sharing (Y/N) per dyad including Social Tolerance**

ASperdyadBINnull: AS ~ Condition + (1 | Group/Subject)

ASperdyadBINst: AS ~ Condition + SocTol + (1 | Group/Subject)

Df AIC BIC logLik deviance Chisq Chi Df

ASperdyadBINnull 5 2530.0 2547.4 -1260.0 2520.0

ASperdyadBINst 6 2490.2 2511.2 -1239.1 2478.2 41.769 1

Pr(>Chisq)

ASperdyadBINnull

ASperdyadBINst 1.027e-10 ***

***Formula: AS ~ Condition + SocTol + (1 | Group/Subject)***

Weights: Weight

AIC BIC logLik deviance df.resid

2490.2 2511.2 -1239.1 2478.2 236

Scaled residuals:

Min 1Q Median 3Q Max

-4.0994 -1.6887 -1.1718 -0.1618 13.8986

Random effects:

Groups Name Variance Std.Dev.

Subject:Group (Intercept) 4.863e+00 2.2051970

Group (Intercept) 1.288e-08 0.0001135

Number of obs: 242, groups: Subject:Group, 10; Group, 2

Fixed effects:

Estimate Std. Error z value Pr(>|z|)

(Intercept) -1.7946 0.7245 -2.477 0.0132 *

ConditionB -0.6148 0.1208 -5.089 3.60e-07 ***

ConditionC -0.7073 0.1275 -5.549 2.88e-08 ***

SocTol -1.7701 0.2811 -6.298 3.01e-10 ***

**Active Sharing (Y/N) per dyad including Sex Combination**

ASperdyadBINnull: AS ~ Condition + (1 | Group/Subject)

ASperdyadBINsc: AS ~ Condition + SEXCombi + (1 | Group/Subject)

Df AIC BIC logLik deviance Chisq Chi Df

ASperdyadBINnull 5 2530.0 2547.4 -1260.0 2520.0

ASperdyadBINsc 8 2414.4 2442.3 -1199.2 2398.4 121.61 3

Pr(>Chisq)

ASperdyadBINnull

ASperdyadBINsc < 2.2e-16 ***

***Formula: AS ~ Condition + SEXCombi + (1 | Group/Subject)***

Weights: Weight

AIC BIC logLik deviance df.resid

2414.4 2442.3 -1199.2 2398.4 234

Scaled residuals:

Min 1Q Median 3Q Max

-3.8420 -1.8037 -0.8447 -0.1381 18.6069

Random effects:

Groups Name Variance Std.Dev.

Subject:Group (Intercept) 2.486e+00 1.577e+00

Group (Intercept) 7.791e-10 2.791e-05

Number of obs: 242, groups: Subject:Group, 10; Group, 2

Fixed effects:

Estimate Std. Error z value Pr(>|z|)

(Intercept) -4.1671 0.7214 -5.776 7.64e-09 ***

ConditionB -0.6287 0.1222 -5.146 2.67e-07 ***

ConditionC -0.7234 0.1290 -5.608 2.05e-08 ***

SEXCombiFM 1.7147 0.2149 7.977 1.49e-15 ***

SEXCombiMF 3.6693 1.0702 3.428 0.000607 ***

SEXCombiMM 2.6291 1.0800 2.434 0.014914 *

$`emmeans of SEXCombi`

SEXCombi emmean SE df asymp.LCL asymp.UCL

FF -4.618 0.720 Inf -6.03 -3.206

FM -2.903 0.701 Inf -4.28 -1.530

MF -0.949 0.792 Inf -2.50 0.604

MM -1.989 0.805 Inf -3.57 -0.410

Results are averaged over the levels of: Condition

Results are given on the logit (not the response) scale.

Confidence level used: 0.95

$`pairwise differences of SEXCombi`

contrast estimate SE df z.ratio p.value

FF - FM -1.715 0.215 Inf -7.977 <.0001

FF - MF -3.669 1.070 Inf -3.428 0.0034

FF - MM -2.629 1.080 Inf -2.434 0.0707

FM - MF -1.955 1.057 Inf -1.849 0.2506

FM - MM -0.914 1.067 Inf -0.857 0.8270

MF - MM 1.040 0.178 Inf 5.829 <.0001

Results are averaged over the levels of: Condition

Results are given on the log odds ratio (not the response) scale.

P value adjustment: tukey method for comparing a family of 4 estimates

**Active Sharing (Y/N) per dyad including Sex Combination*Social Tolerance**

ASperdyadBINnull: AS ~ Condition + (1 | Group/Subject)

ASperdyadBINint: AS ~ Condition + SEXCombi:SocTol + (1 | Group/Subject)

Df AIC BIC logLik deviance Chisq Chi Df

ASperdyadBINnull 5 2530.0 2547.4 -1260.0 2520.0

ASperdyadBINint 9 2359.1 2390.5 -1170.6 2341.1 178.88 4

Pr(>Chisq)

ASperdyadBINnull

ASperdyadBINint < 2.2e-16 ***

***Formula: AS ~ Condition + SEXCombi:SocTol + (1 | Group/Subject)***

Weights: Weight

AIC BIC logLik deviance df.resid

2359.1 2390.5 -1170.6 2341.1 233

Scaled residuals:

Min 1Q Median 3Q Max

-4.2205 -1.7490 -0.8394 -0.1231 14.5073

Random effects:

Groups Name Variance Std.Dev.

Subject:Group (Intercept) 5.070e+00 2.2517386

Group (Intercept) 8.052e-08 0.0002838

Number of obs: 242, groups: Subject:Group, 10; Group, 2

Fixed effects:

Estimate Std. Error z value Pr(>|z|)

(Intercept) -1.7732 0.7413 -2.392 0.0167 *

ConditionB -0.6403 0.1233 -5.193 2.07e-07 ***

ConditionC -0.7373 0.1302 -5.662 1.49e-08 ***

SEXCombiFF:SocTol -11.9118 2.9531 -4.034 5.49e-05 ***

SEXCombiFM:SocTol 0.4250 0.5047 0.842 0.3997

SEXCombiMF:SocTol -1.7548 0.3950 -4.442 8.90e-06 ***

SEXCombiMM:SocTol -4.8152 0.6116 -7.873 3.46e-15 ***

**Begging**

***First hurdle: likelihood of begging***

BEGBINnull: BEGBIN ~ (1 | Group/Individual)

BEGBIN: BEGBIN ~ Condition + Sex + Condition * Sex + (1 | Group/Individual)

Df AIC BIC logLik deviance Chisq Chi Df

BEGBINnull 3 828.91 835.04 -411.45 822.91

BEGBIN 8 606.94 623.29 -295.47 590.94 231.96 5

Pr(>Chisq)

BEGBINnull

BEGBIN < 2.2e-16***

***Formula: BEGBIN ~ Condition + Sex + Condition * Sex + (1 | Group/Individual)***

Weights: Weight

AIC BIC logLik deviance df.resid

606.9 623.3 -295.5 590.9 49

Scaled residuals:

Min 1Q Median 3Q Max

-11.8953 -1.5555 -0.0536 1.2550 8.8622

Random effects:

Groups Name Variance Std.Dev.

Individual:Group (Intercept) 15.230 3.903

Group (Intercept) 2.949 1.717

Number of obs: 57, groups: Individual:Group, 10; Group, 2

Fixed effects:

Estimate Std. Error z value Pr(>|z|)

(Intercept) 0.87636 2.09757 0.418 0.676

ConditionB -3.90936 0.35961 -10.871 <2e-16 ***

ConditionC -2.51900 0.27966 -9.007 <2e-16 ***

SexM -0.54561 2.76196 -0.198 0.843

ConditionB:SexM 0.07912 1.35407 0.058 0.953

ConditionC:SexM -1.33268 1.33498 -0.998 0.318

***Second hurdle: number of begging calls when begging occurred***

BEGnull: BEG ~ (1 | Group/Individual)

BEG: BEG ~ Condition + Sex + Sex * Condition + (1 | Group/Individual)

Df AIC BIC logLik deviance Chisq Chi Df Pr(>Chisq)

BEGnull 4 -6194.3 -6189.9 3101.1 -6202.3

BEG 9 -3356.0 -3346.2 1687.0 -3374.0 0 5 1

***Formula: BEG ~ Condition + Sex + Sex * Condition + (1 | Group/Individual)***

Data: subset(xdata, BEG > 0)

Weights: Weight

AIC BIC logLik deviance df.resid

-3356.0 -3346.2 1687.0 -3374.0 13

Scaled residuals:

Min 1Q Median 3Q Max

23.82 47.73 77.91 210.85 673.57

Random effects:

Groups Name Variance Std.Dev.

Individual:Group (Intercept) 0 0

Group (Intercept) 0 0

Number of obs: 22, groups: Individual:Group, 8; Group, 2

Fixed effects:

Estimate Std. Error z value Pr(>|z|)

(Intercept) -3.746510 0.135666 -27.616 <2e-16 ***

ConditionB 0.031831 0.299531 0.106 0.915

ConditionC -0.146239 0.479658 -0.305 0.760

SexM 0.025116 0.202867 0.124 0.901

ConditionB:SexM -0.061008 0.414439 -0.147 0.883

ConditionC:SexM 0.005951 0.719626 0.008 0.993

**Total Sharing (TS; i.e. both active and passive sharing) including Begging**

***First Hurdle: Likelihood***

TSBEGbinnull: TSBIN ~ (1 | Group/Individual)

TSBEGbinom: TSBIN ~ BEG + BEG:Condition + (1 | Group/Individual)

Df AIC BIC logLik deviance Chisq Chi Df

TSBEGbinnull 3 811.56 817.69 -402.78 805.56

TSBEGbinom 6 788.88 801.14 -388.44 776.88 28.686 3

Pr(>Chisq)

TSBEGbinnull

TSBEGbinom 2.607e-06 ***

***Formula: TSBIN ~ BEG + BEG:Condition + (1 | Group/Individual)***

Weights: Weight

AIC BIC logLik deviance df.resid

788.9 801.1 -388.4 776.9 51

Scaled residuals:

Min 1Q Median 3Q Max

-9.2891 -2.6752 0.1813 2.7371 5.2812

Random effects:

Groups Name Variance Std.Dev.

Individual:Group (Intercept) 1.288e+01 3.589e+00

Group (Intercept) 1.674e-09 4.091e-05

Number of obs: 57, groups: Individual:Group, 10; Group, 2

Fixed effects:

Estimate Std. Error z value Pr(>|z|)

(Intercept) 0.98524 1.16992 0.842 0.39971

BEG 0.05988 0.02501 2.395 0.01664 *

BEG:ConditionB -0.32415 0.11204 -2.893 0.00382 **

BEG:ConditionC 0.16531 0.20586 0.803 0.42196

$`emmeans of BEG, Condition`

BEG Condition emmean SE df asymp.LCL asymp.UCL

2.58 A 1.140 1.17 Inf -1.152 3.43

2.58 B 0.304 1.18 Inf -2.000 2.61

2.58 C 1.566 1.27 Inf -0.932 4.06

Results are given on the logit (not the response) scale.

Confidence level used: 0.95

$`pairwise differences of BEG, Condition`

contrast estimate SE df z.ratio

2.57894736842105,A - 2.57894736842105,B 0.836 0.289 Inf 2.893

2.57894736842105,A - 2.57894736842105,C -0.426 0.531 Inf -0.803

2.57894736842105,B - 2.57894736842105,C -1.262 0.596 Inf -2.118

p.value

0.0107

0.7011

0.0863

Results are given on the log odds ratio (not the response) scale.

P value adjustment: tukey method for comparing a family of 3 estimates

***Second Hurdle: Number of sharing events when food-sharing occurred***

TSBEGnull: TS ~ (1 | Group/Individual)

TSBEG: TS ~ BEG + Condition:BEG + (1 | Group/Individual)

Df AIC BIC logLik deviance Chisq Chi Df Pr(>Chisq)

TSBEGnull 4 468.9 474.2 -230.5 460.9

TSBEG 7 7691.9 7701.2 -3839.0 7677.9 0 3 1

***Formula: FAS ~ BEG + Condition:BEG + (1 | Group/Individual)***

Data: subset(xdata1, FAS > 0)

Weights: Weight

AIC BIC logLik deviance df.resid

6263.1 6272.4 -3124.5 6249.1 21

Scaled residuals:

Min 1Q Median 3Q Max

10.11 10.74 22.69 44.85 154.15

Random effects:

Groups Name Variance Std.Dev.

Individual:Group (Intercept) 0 0

Group (Intercept) 0 0

Number of obs: 28, groups: Individual:Group, 9; Group, 2

Fixed effects:

Estimate Std. Error z value Pr(>|z|)

(Intercept) -2.28614 0.13816 -16.547 <2e-16 ***

BEG 0.01471 0.01346 1.093 0.275

BEG:ConditionB -0.04065 0.06708 -0.606 0.545

BEG:ConditionC 0.07945 0.14379 0.553 0.581

**Active Sharing (FAS) including Begging**

***First Hurdle: Likelihood***

AFSBEGbinnull: FASBIN ~ (1 | Group/Individual)

AFSBEGbinom: FASBIN ~ Condition + BEG:Condition + (1 | Group/Individual)

Df AIC BIC logLik deviance Chisq Chi Df

AFSBEGbinnull 3 835.46 841.59 -414.73 829.46

AFSBEGbinom 8 757.56 773.90 -370.78 741.56 87.903 5

Pr(>Chisq)

AFSBEGbinnull

AFSBEGbinom < 2.2e-16 ***

***Formula: FASBIN ~ Condition + BEG:Condition + (1 | Group/Individual)***

Weights: Weight

AIC BIC logLik deviance df.resid

757.6 773.9 -370.8 741.6 49

Scaled residuals:

Min 1Q Median 3Q Max

-7.9592 -2.1022 -0.1724 2.1587 8.1947

Random effects:

Groups Name Variance Std.Dev.

Individual:Group (Intercept) 1.689e+01 4.1095872

Group (Intercept) 4.647e-08 0.0002156

Number of obs: 57, groups: Individual:Group, 10; Group, 2

Fixed effects:

Estimate Std. Error z value Pr(>|z|)

(Intercept) 1.77870 1.34460 1.323 0.1859

ConditionB -1.61867 0.25646 -6.312 2.76e-10 ***

ConditionC -1.54861 0.27753 -5.580 2.40e-08 ***

ConditionA:BEG -0.04387 0.02940 -1.492 0.1357

ConditionB:BEG -0.26938 0.11746 -2.293 0.0218 *

ConditionC:BEG -0.34690 0.21895 -1.584 0.1131

$`emmeans of BEG, Condition`

BEG Condition emmean SE df asymp.LCL asymp.UCL

2.58 A 1.666 1.34 Inf -0.96 4.29

2.58 B -0.535 1.34 Inf -3.17 2.10

2.58 C -0.665 1.42 Inf -3.45 2.12

Results are given on the logit (not the response) scale.

Confidence level used: 0.95

$`pairwise differences of BEG, Condition`

contrast estimate SE df z.ratio

2.57894736842105,A - 2.57894736842105,B 2.20 0.369 Inf 5.970

2.57894736842105,A - 2.57894736842105,C 2.33 0.540 Inf 4.314

2.57894736842105,B - 2.57894736842105,C 0.13 0.596 Inf 0.218

p.value

<.0001

<.0001

0.9742

Results are given on the log odds ratio (not the response) scale.

P value adjustment: tukey method for comparing a family of 3 estimates

***Second Hurdle: Number of sharing events when food-sharing occurred***

AFSBEGnull: FAS ~ (1 | Group/Individual)

AFSBEG: FAS ~ BEG + Condition:BEG + (1 | Group/Individual)

Df AIC BIC logLik deviance Chisq Chi Df Pr(>Chisq)

AFSBEGnull 4 123.7 129.1 -57.86 115.7

AFSBEG 7 6263.1 6272.4 -3124.53 6249.1 0 3 1

***Formula: FAS ~ BEG + Condition:BEG + (1 | Group/Individual)***

Data: subset(xdata1, FAS > 0)

Weights: Weight

AIC BIC logLik deviance df.resid

6263.1 6272.4 -3124.5 6249.1 21

Scaled residuals:

Min 1Q Median 3Q Max

10.11 10.74 22.69 44.85 154.15

Random effects:

Groups Name Variance Std.Dev.

Individual:Group (Intercept) 0 0

Group (Intercept) 0 0

Number of obs: 28, groups: Individual:Group, 9; Group, 2

Fixed effects:

Estimate Std. Error z value Pr(>|z|)

(Intercept) -2.28614 0.13816 -16.547 <2e-16 ***

BEG 0.01471 0.01346 1.093 0.275

BEG:ConditionB -0.04065 0.06708 -0.606 0.545

BEG:ConditionC 0.07945 0.14379 0.553 0.581

**Additional Analyses on other call types**

**#Contact Calls#**

ContactCallsnull: CONT ~ (1 | Group/Individual)

ContactCalls: CONT ~ Condition + Sex + Sex * Condition + (1 | Group/Individual)

Df AIC BIC logLik deviance Chisq Chi Df

ContactCallsnull 4 578.01 586.18 -285.01 570.01

ContactCalls 9 577.81 596.20 -279.91 559.81 10.2 5

Pr(>Chisq)

ContactCallsnull

ContactCalls 0.06976

***Formula: CONT ~ Condition + Sex + Sex * Condition + (1 | Group/Individual)***

Weights: Weight

REML criterion at convergence: 518.3

Scaled residuals:

Min 1Q Median 3Q Max

-1.8535 -0.8110 -0.0552 0.5201 3.3465

Random effects:

Groups Name Variance Std.Dev.

Individual:Group (Intercept) 50.57 7.111

Group (Intercept) 313.61 17.709

Residual 15892.58 126.066

Number of obs: 57, groups: Individual:Group, 10; Group, 2

Fixed effects:

Estimate Std. Error df t value Pr(>|t|)

(Intercept) 61.9554 15.9987 1.9581 3.873 0.06276 .

ConditionB 11.2448 13.7833 42.9526 0.816 0.41910

ConditionC 36.7699 13.4043 42.4763 2.743 0.00887 **

SexM -4.4554 15.6279 28.8758 -0.285 0.77761

ConditionB:SexM -0.6198 21.3274 42.6340 -0.029 0.97695

ConditionC:SexM -17.0714 22.1318 43.2253 -0.771 0.44470

**#Soft Calls#**

SoftCallsnull: SC ~ (1 | Group/Individual)

SoftCalls: SC ~ Condition + Sex + Sex * Condition + (1 | Group/Individual)

Df AIC BIC logLik deviance Chisq Chi Df

SoftCallsnull 4 551.98 560.16 -271.99 543.98

SoftCalls 9 553.53 571.92 -267.76 535.53 8.4558 5

Pr(>Chisq)

SoftCallsnull

SoftCalls 0.1328

***Formula: SC ~ Condition + Sex + Sex * Condition + (1 | Group/Individual)***

Weights: Weight

REML criterion at convergence: 497.1

Scaled residuals:

Min 1Q Median 3Q Max

-1.86899 -0.71427 -0.06212 0.50870 1.73834

Random effects:

Groups Name Variance Std.Dev.

Individual:Group (Intercept) 104.51 10.223

Group (Intercept) 67.18 8.196

Residual 10079.89 100.399

Number of obs: 57, groups: Individual:Group, 10; Group, 2

Fixed effects:

Estimate Std. Error df t value Pr(>|t|)

(Intercept) 56.941 10.419 3.376 5.465 0.00879 **

ConditionB 10.073 10.987 43.581 0.917 0.36429

ConditionC 7.845 10.676 43.264 0.735 0.46645

SexM -15.191 13.605 23.741 -1.117 0.27536

ConditionB:SexM 11.802 16.991 43.371 0.695 0.49102

ConditionC:SexM 29.501 17.651 43.736 1.671 0.10180

**R-code**

#Libraries used#

library(lme4)

library(lmerTest)

library(car)

library(MASS)

library(fitdistrplus)

library(survival)

library(fitdistrplus)

library(robustlmm)

library(arm)

library(MASS)

library(Matrix)

library(pscl)

library(MuMIn)

library(pbkrtest)

library(emmeans)

library(irr)

library(ggplot2)

#CASHE distribution accross condition and sex#

CASHE<-lmer(CACHE~Condition+Sex+Condition*Sex+(1|Group/Individual), data= xdata, weights = Weight)

CASHEnull<-lmer(CACHE~(1|Group/Individual), data= xdata, weights = Weight)

anova(CASHE,CASHEnull)

summary(CASHE)

#Hurdle model on TS (Active Food Sharing + Passive Food Sharing)#

#first hurdle#

TSbinom <- glmer(TSBIN~Condition+Sex+Condition*Sex+(1|Group/Individual), data= xdata, weights = Weight, family = "binomial")

TSbinnull <- glmer(TSBIN~(1|Group/Individual), data= xdata, weights = Weight, family = "binomial")

anova(TSbinom,TSbinnull)

summary(TSbinom)

anova(TSbinom)

emmeans(TSbinom, list(pairwise ~ Sex*Condition))

#second hurdle#

TS <- glmer.nb(TS~Condition+Sex+Sex*Condition+(1|Group/Individual),

data=subset(xdata,TS>0), weights = Weight)

TSnull <- glmer.nb(TS~(1|Group/Individual),

data=subset(xdata,TS>0), weights = Weight)

anova(TS,TSnull)

summary(TS)

#Hurdle model on AFS (Active Food Sharing)#

#first hurdle#

AFSbinom <- glmer(FASBIN~Condition+Sex+Condition*Sex+(1|Group/Individual), data= xdata, weights = Weight, family = "binomial")

AFSbinnull <- glmer(FASBIN~(1|Group/Individual), data= xdata, weights = Weight, family = "binomial")

anova(AFSbinom, AFSbinnull)

summary(AFSbinom)

emmeans(AFSbinom, list(pairwise ~ Sex*Condition))

#second hurdle#

AFS <- glmer.nb(FAS~Condition+Sex+Sex*Condition+(1|Group/Individual),

data=subset(xdata,FAS>0), weights = Weight)

AFSnull <- glmer.nb(FAS~(1|Group/Individual),

data=subset(xdata,FAS>0), weights = Weight)

anova(AFS,AFSnull)

summary(AFS)

#Sharing and Dyadic Characteristics#

#Total Sharing#

xdata1<-read.csv(file.choose())

fitSocialTolerance <- lmer(SocTol~SEXCombi+(1|Group/Subject), data = xdata1)

anova(fitSocialTolerance)

emmeans(fitSocialTolerance, list(pairwise ~ SEXCombi), adjust = "tukey")

TSperdyadBINst <- glmer(TSBIN~Condition+SocTol+(1|Group/Subject), data= xdata1, weights = Weight, family = "binomial")

summary(TSperdyadBINst)

TSperdyadBINnull <- glmer(TSBIN~Condition+(1|Group/Subject), data= xdata1, weights = Weight, family = "binomial")

anova(TSperdyadBINst,TSperdyadBINnull)

TSperdyadBINsc <- glmer(TSBIN~Condition+SEXCombi+(1|Group/Subject), data= xdata1, weights = Weight, family = "binomial")

summary(TSperdyadBINsc)

anova(TSperdyadBINsc,TSperdyadBINnull)

emmeans(TSperdyadBINsc, list(pairwise ~ SEXCombi), adjust = "tukey")

TSperdyadBINint <- glmer(TSBIN~Condition+SEXCombi:SocTol+(1|Group/Subject), data= xdata1, weights = Weight, family = "binomial")

summary(TSperdyadBINint)

anova(TSperdyadBINint,TSperdyadBINnull)

#Active Sharing#

ASperdyadBINst <- glmer(AS~Condition+SocTol+(1|Group/Subject), data= xdata1, weights = Weight, family = "binomial")

summary(ASperdyadBINst)

ASperdyadBINnull <- glmer(AS~Condition+(1|Group/Subject), data= xdata1, weights = Weight, family = "binomial")

anova(ASperdyadBINst,ASperdyadBINnull)

ASperdyadBINsc <- glmer(AS~Condition+SEXCombi+(1|Group/Subject), data= xdata1, weights = Weight, family = "binomial")

summary(ASperdyadBINsc)

anova(ASperdyadBINsc,ASperdyadBINnull)

emmeans(ASperdyadBINsc, list(pairwise ~ SEXCombi), adjust = "tukey")

ASperdyadBINint <- glmer(AS~Condition+SEXCombi:SocTol+(1|Group/Subject), data= xdata1, weights = Weight, family = "binomial")

summary(ASperdyadBINint)

anova(ASperdyadBINint,ASperdyadBINnull)

#Hurdle model on BEG (Begging)#

#first hurdle#

BEGBIN <- glmer(BEGBIN~Condition+Sex+Condition*Sex+(1|Group/Individual), data= xdata, weights = Weight, family = "binomial")

BEGBINnull <- glmer(BEGBIN~(1|Group/Individual), data= xdata, weights = Weight, family = "binomial")

anova(BEGBIN,BEGBINnull)

summary(BEGBIN)

#second hurdle#

BEG <- glmer.nb(BEG~Condition+Sex+Sex*Condition+(1|Group/Individual),

data=subset(xdata,BEG>0), weights = Weight)

BEGnull <- glmer.nb(BEG~(1|Group/Individual),

data=subset(xdata,BEG>0), weights = Weight)

anova(BEG,BEGnull)

summary(BEG)

#Contact Calls#

ContactCalls <- lmer(CONT~Condition+Sex+Sex*Condition+(1|Group/Individual),

data=xdata, weights = Weight)

ContactCallsnull <- lmer(CONT~(1|Group/Individual),

data=xdata, weights = Weight)

anova(ContactCalls,ContactCallsnull)

summary(ContactCalls)

ggplot(data=xdata, aes(x=Condition, y=CONT, fill=Condition)) +

geom_boxplot(outlier.colour="black", outlier.shape=16,

outlier.size=2, notch=FALSE) + coord_cartesian(ylim = c(0, 200))

#Soft Calls#

SoftCalls <- lmer(SC~Condition+Sex+Sex*Condition+(1|Group/Individual),

data=xdata, weights = Weight)

SoftCallsnull <- lmer(SC~(1|Group/Individual),

data=xdata, weights = Weight)

anova(SoftCalls,SoftCallsnull)

summary(SoftCalls)

#Hurdle model on AFS (Active Food Sharing), including begging#

#first hurdle#

xdata1<-read.csv(file.choose())

TSBEGbinom <- glmer(TSBIN~BEG+BEG:Condition+(1|Group/Individual), data= xdata1, weights = Weight, family = "binomial")

TSBEGbinnull <- glmer(TSBIN~(1|Group/Individual), data= xdata1, weights = Weight, family = "binomial")

anova(TSBEGbinom, TSBEGbinnull)

summary(TSBEGbinom)

emmeans(TSBEGbinom, list(pairwise ~ BEG:Condition), adjust = "tukey")

AFSBEGbinom <- glmer(FASBIN~Condition+BEG:Condition+(1|Group/Individual), data= xdata1, weights = Weight, family = "binomial")

AFSBEGbinnull <- glmer(FASBIN~(1|Group/Individual), data= xdata1, weights = Weight, family = "binomial")

anova(AFSBEGbinom, AFSBEGbinnull)

summary(AFSBEGbinom)

emmeans(AFSBEGbinom, list(pairwise ~ BEG:Condition), adjust = "tukey")

#second hurdle#

TSBEG <- glmer.nb(TS~BEG+Condition:BEG+(1|Group/Individual),

data=subset(xdata1,FAS>0), weights = Weight)

TSBEGnull <- glmer.nb(TS~(1|Group/Individual),

data=subset(xdata1,FAS>0), weights = Weight)

anova(TSBEG,TSBEGnull)

summary(AFSBEG)

AFSBEG <- glmer.nb(FAS~BEG+Condition:BEG+(1|Group/Individual),

data=subset(xdata1,FAS>0), weights = Weight)

AFSBEGnull <- glmer.nb(FAS~(1|Group/Individual),

data=subset(xdata1,FAS>0), weights = Weight)

anova(AFSBEG,AFSBEGnull)

summary(AFSBEG)
